# Research on microgrid cluster optimization method based on sparrow search algorithm

**DOI:** 10.1038/s41598-026-48617-w

**Published:** 2026-04-14

**Authors:** Hongzhi Su, Pengtao Mu, Shenglin Xu, Lingrao Wang, Liying Yu, Bo Zhang

**Affiliations:** North China Branch of State Grid Corporation of China, Beijing, 100053 China

**Keywords:** Microgrid cluster, Distributed generation, Economic optimization model, Sparrow search algorithm, Energy science and technology, Engineering, Mathematics and computing

## Abstract

With the widespread application of renewable energy in microgrids, collaborative optimization scheduling of microgrid clusters has become a key issue in improving energy utilization efficiency and operational economy. To solve this problem, this paper proposes a microgrid cluster optimization scheduling method based on the sparrow search algorithm. Firstly, construct a microgrid cluster model that includes wind turbines, photovoltaics, energy storage batteries, diesel generators, and hydrogen fuel cells. Secondly, the economic optimum is defined as the objective function, and combined with the constraints of equipment and system operation, the sparrow search algorithm is proposed to solve the optimization model of the microgrid cluster. Finally, this method is used to simulate an IEEE 9-node system with 4 microgrids. The simulation results showed that this method has significant advantages over traditional particle swarm optimization algorithms in reducing total costs and new energy utilization efficiency, verifying the feasibility of this method in microgrid cluster optimization scheduling.

## Introduction

A microgrid is a controllable and independent source-grid-load-storage system. It comprises loads, monitoring and protection equipment, and distributed generations (DGs) such as photovoltaics (PVs), wind turbines (WTs), diesel generators, and energy storage devices^1,2^. Microgrid can automatically manage and allocate energy based on changes in load and power availability, thereby improving the reliability and safety of power supply in the power grid^3–6^. Because WTs and PVs can utilize renewable energy to generate electricity, the direction of shifting from fossil fuels, coal, and other fuels to renewable energy has been achieved, improving the utilization rate of clean energy and reducing environmental pollution^7^. Under certain conditions, the microgrid can achieve self-sufficiency in electric energy or operate independently without relying on a large power grid, reducing dependence on the power system^8–10^. Due to the diversity of DGs, users can flexibly choose the source of electricity according to their own needs, which can reduce the cost of electricity transmission and achieve sustainable development of the environment and society. These have broad application prospects.

Although microgrid has reliable and flexible power supply characteristics, individual microgrid has disadvantages such as poor anti-interference ability and limited capacity. Thus, the concept of a microgrid cluster was proposed^11^, which consists of multiple independent microgrid systems with strong anti-interference ability. It can achieve energy exchange with the large grid and also exchange energy between internal microgrids^12,13^, which is a trend in microgrid development^14^. However, the economic optimization of a microgrid cluster is more complex than that of individual microgrids. It has become a hot research topic both domestically and internationally. Such as^15^, considering both inter-microgrid power scheduling and the economic benefits of power procurement, a multi-objective optimization approach for energy scheduling is proposed based on the Non-dominated Sorting Genetic Algorithm II (NSGA-II) and the Constraint-based Multi-objective Evolutionary Algorithm based on Decomposition (CMOEA/D). However, the application scenarios of the paper are relatively fixed, and there are still real-time challenges when dealing with larger scale clusters in multi-objective evolutionary algorithms. In^16^, with the goal of minimizing the total operating cost of the distribution network-microgrid cluster system, a leader-follower game model for the economic dispatch of distribution network-microgrid clusters is established. An emergent revenue allocation method is proposed based on the value of mutual aid power. The paper adopts the K-means clustering algorithm for sub microgrid stratification. Although this method has high computational efficiency, K-means is sensitive to the initial clustering center and requires a predetermined number of clusters *k*. In practical applications, the optimal number of levels may dynamically change over time, and how to adaptively determine the number of clusters still needs to be studied. In^17^, a low-carbon economic scheduling model for the microgrid cluster is constructed based on model predictive control (MPC). A distributed multiple time-scales energy management strategy for islanded microgrid clusters is proposed, which can enhance the accuracy and feasibility of scheduling plans by coordinating long and short time scales. However, the research focuses on islanded mode microgrid clusters, which is applicable to remote areas or special scenarios and limits the promotion of the strategy in grid connected microgrids. In^18^, to effectively reduce the microgrid cluster’s operating costs and power fluctuations and achieve mutual benefits for the microgrids and the shared energy storage, the paper proposes a multi-time-scale game dispatching strategy of the shared energy storage and the microgrids with the uncertainty of demand response. However, nested games with multiple time scales and uncertainty modeling lead to high model dimensions and difficult solutions, especially when applied in large-scale clusters. The convergence speed and computational efficiency of the algorithm still need to be discussed.

With the advancement of the electricity market, microgrid clusters play an important role in electricity market transactions^19^. The microgrid clusters contain more distributed power sources. To achieve the financial operation of microgrid clusters under relevant constraints, it is necessary to consider the supply relationships of multiple energy forms and the electricity prices of the large grid at different times. The optimal control of microgrid clusters is relatively tricky. Thus, this paper proposes a microgrid cluster economic optimization scheduling method based on a sparrow search algorithm to solve this issue. Firstly, models of DGs such as WT, PV, diesel generators, storage batteries, and hydrogen fuel cells are constructed separately. Secondly, the objective function of economic optimization is to minimize the total cost composed of the operating costs of each micro power source, transaction costs between microgrids and large grids, and mutual transaction costs between microgrids. The sparrow search algorithm is used to solve the problem. Finally, a microgrid cluster system consisting of four individual microgrids was constructed on the IEEE9 system. By comparing it with the particle swarm optimization algorithm, the feasibility and correctness of the method are verified.

In this context, this study proposes an optimal method for the microgrid cluster based on the sparrow search algorithm. The main contributions of this paper are summarized as follows:

i. Introducing the models of DGs such as WT, PV, diesel generators, storage batteries, and hydrogen fuel cells.
ii. Defining the objective function for optimization and the constraints for system operation.
iii. Taking the sparrow search algorithm to solve the optimization model and obtain the optimal operation result of the microgrid cluster.

The remainder of this paper is organized as follows: Sect. 2 introduces the WT, PV, diesel generators, storage batteries, and hydrogen fuel cells models. Section 3 defines the economic optimization model and constraints. Section 4 introduces the sparrow search algorithm and solves the optimization model. Section 5 presents case studies to demonstrate the feasibility of the proposed approach. Finally, Sect. 6 concludes the paper.

##  DG model

1. WT model.
  WT is a device that can convert wind energy into electrical energy. The relationship between output power and wind speed is as follows:
  1 $${P^{\prime}_{WT}}(t)=\left\{ {\begin{array}{*{20}{c}} 0&{0<v<{{v^{\prime}}_{ci}}} \\ {P_{r}^{\prime } \times \frac{{v - {{v^{\prime}}_{ci}}}}{{{{v^{\prime}}_r} - {{v^{\prime}}_{ci}}}}}&{{{v^{\prime}}_{ci}} \leqslant v \leqslant v^{\prime}} \\ {P_{r}^{\prime }}&{{{v^{\prime}}_r} \leqslant v \leqslant {{v^{\prime}}_{co}}} \\ 0&{v \geqslant {{v^{\prime}}_{co}}} \end{array}} \right.$$
2. PV model.
  The principle of PV is to convert solar energy into electrical energy, and its output power is:
  2 $${P^{\prime}_{pv}}(t)={R^{\prime}_{pv}}{q^{\prime}_{pv}}\frac{{{{I^{\prime}}_T}(t)}}{{{{I^{\prime}}_{STC}}}}\left[ {1+{{\alpha ^{\prime}}_p}\left( {{{T^{\prime}}_c}(t) - {{T^{\prime}}_{stc}}} \right)} \right]$$
  Where ${q^{\prime}_{pv}}$ can be taken as 0.8.
3. Energy storage battery model.
  As an energy storage device in microgrids, it can ensure the continuous power supply when WT and PV occur intermittent power supply, which enhances the power supply reliability of the microgrid. The commonly used State of Charge (SOC) represents the amount of remaining charge, which is:
  3 $$SOC(t)=\left\{ {\begin{array}{*{20}{c}} {SOC(t - 1)+\frac{1}{{{\eta ^ - }}}{P_{bess}}(t)}&{{P_{bess}} \leqslant 0} \\ {SOC(t - 1)+\frac{1}{{{\eta ^+}}}{P_{bess}}(t)}&{{P_{bess}}>0} \end{array}} \right.$$
  When ${P_{bess}}(t)>0$, the energy storage battery is charging, when ${P_{bess}}(t) \leqslant 0$, the energy storage battery is discharging.
4. Diesel generator model
  4 $${P_{{\mathrm{gen}}}}(t)={\eta _{xl}} \times {\dot {m}_f} \times {Q_{LHV}}$$
  Where ${\eta _{xl}}$ is the overall efficiency; ${\dot {m}_f}$ is the fuel mass flow rate; ${Q_{LHV}}$ is the low calorific value of the fuel.
5. Hydrogen Fuel Cell model
  5 $$\left\{ \begin{gathered} P_{{{H_2},ele}}^{t}=\eta _{{ele}}^{{{H_2}}}P_{{e,ele}}^{t} \hfill \\ P_{{{h_e},ele}}^{t}=\eta _{{ele}}^{{{h_e}}}(1 - \eta _{{ele}}^{{{H_2}}})P_{{e,ele}}^{t} \hfill \\ P_{{e,fc}}^{t}=\eta _{{fc}}^{e}P_{{{H_2},fc}}^{t} \hfill \\ P_{{{h_e},fc}}^{t}=\eta _{{fc}}^{{he}}(1 - \eta _{{fc}}^{e})P_{{{H_2},fc}}^{t} \hfill \\ E_{{ht}}^{t}=E_{{ht}}^{{t - 1}}+\smallint ({\eta _{cha}}P_{{{H_2}}}^{{cha}} - \frac{{P_{{{H_2}}}^{{dis}}}}{{{\eta _{dis}}}})dt \hfill \\ \end{gathered} \right.$$

## Economic optimization model and constraints

### Economic optimization model

The objective function mainly consists of three parts: the operating cost of each microgrid, the transaction cost between microgrids and the power system, and the mutual transaction cost between microgrids, as follows:

6 $$F=\hbox{min} \sum\limits_{{i=1}}^{N} {f_{{DE}}^{i}} +f_{m}^{i}+f_{{FC}}^{i}+f_{s}^{i}+f_{{el}}^{i}+f_{{buy}}^{i} - f_{{sell}}^{i}$$

And

7 $$f_{{DE}}^{i}=\sum\limits_{{j=1}}^{{{N_{DE}}}} {\sum\limits_{{t=1}}^{T} {u_{{DE}}^{{j.t}}({\alpha _j}P_{{{\mathrm{DG}}j}}^{2}(t)+{\beta _j}{P_{{\mathrm{DG}}j}}(t)+{\gamma _j})} }$$

8 $$f_{m}^{i}=\sum\limits_{{j=1}}^{{{N_{DE}}}} {\sum\limits_{{t=1}}^{T} {{a_{m,j}}} } *P_{{DG}}^{{j,t}}$$

9 $$f_{{FC}}^{i}=\sum\limits_{{j=1}}^{{{N_{FC}}}} {\sum\limits_{{t=1}}^{T} {\frac{{{C_{ch4}}}}{{LHV}}} } *\frac{{P_{{FC}}^{{j,t}}}}{{ - 0.0023P_{{FC}}^{{j,t}}+0.6735}}$$

10 $$f_{s}^{i}=\sum\limits_{{j=1}}^{{{N_{DE}}}} {{K_{DE}}*n_{{DE}}^{j}}$$

11 $$\begin{gathered} f_{{buy}}^{i}=\sum\limits_{{t=1}}^{T} {q_{{grid - MGi}}^{t}*P_{{grid - MGi}}^{t}} \\ +\sum\limits_{{k=1,k \ne i}}^{N} {\sum\limits_{{t=1}}^{T} {q_{{MGk - MGi}}^{t}*P_{{MGk - MGi}}^{t}} } \\ \end{gathered}$$

12 $$\begin{gathered} f_{{sell}}^{i}=\sum\limits_{{t=1}}^{T} {q_{{MGi - grid}}^{t}*P_{{MGi - grid}}^{t}} \\ +\sum\limits_{{k=1,k \ne i}}^{N} {\sum\limits_{{t=1}}^{T} {q_{{MGi - MGk}}^{t}*P_{{MGi - MGk}}^{t}} } \\ \end{gathered}$$

13 $$f_{{{\mathrm{el}}}}^{i}=\sum\limits_{{j=1}}^{{{N_{el}}}} {\sum\limits_{{t=1}}^{T} {\frac{{P_{{el}}^{{j,t}}*13}}{{33.3}}} }$$

Where *LHV* is the natural gas geothermal heating value, the typical range of natural gas calorific value in Chinese urban areas is 9.7 ~ 15.28kWh/m³, this paper takes 9.7 kWh/m^3^.

### Constraints

1. Energy conservation means that the energy exchanged between microgrids and the power grid, the electricity generated by distributed power sources, the electricity exchanged between microgrids, and the electricity consumption of users in microgrids must be balanced at all times.
  14 $$\sum\limits_{{i=1}}^{N} {\sum\limits_{{j=1}}^{{{N_{{\mathrm{DG}}}}}} {P_{{{\mathrm{DG}}}}^{{i,j,t}}+} P_{{grid}}^{t}} =P_{{load}}^{t}+P_{{loss}}^{t}$$
2. The upper and lower limits of the output of DGs are as follows:
  15 $$P_{{DG}}^{{i,j,\hbox{min} }} \leqslant B_{{{\mathrm{DG}}}}^{{i,j,t}}P_{{DG}}^{{i,j,t}} \leqslant P_{{DG}}^{{i,j,\hbox{max} }}$$
  Where $B_{{{\mathrm{DG}}}}^{{i,j,t}}$ is the state of the DG *j* at time *t* in microgrid *i*,1 is running, 0 is stopped.
3. Generator climbing constraint
  16 $$- P_{{i,genj,down}}^{{\hbox{max} }} \leqslant B_{{i,genj}}^{t}(P_{{i,genj}}^{t} - P_{{i,genj}}^{{t - 1}}) \leqslant P_{{i,genj,up}}^{{\hbox{max} }}$$
  Where $B_{{i,genj}}^{t}$ is the state of the generator *j* in microgrid *i* at time *t*,1 is running, 0 is stopped.
4. Battery operation constraints
  17 $$\left\{ \begin{gathered} E_{{_{{\hbox{min} }}}}^{{i,j}} \leqslant {E^{i,j,t}} \leqslant E_{{\hbox{max} }}^{{i,j}} \hfill \\ 0 \leqslant K_{{ch\arg e}}^{{i,j,t}}P_{{ch\arg e}}^{{i,j,t}} \leqslant P_{{ch\arg e}}^{{i,j,t\hbox{max} }} \hfill \\ 0 \leqslant K_{{disch\arg e}}^{{i,j,t}}P_{{disch\arg e}}^{{i,j,t}} \leqslant P_{{disch\arg e}}^{{i,j,t\hbox{max} }} \hfill \\ SOC_{{\hbox{min} }}^{{i,j}} \leqslant SO{C^{i,j,t}} \leqslant SOC_{{\hbox{max} }}^{{i,j}} \hfill \\ DOD_{{\hbox{min} }}^{{i,j}} \leqslant DO{D^{i,j,t}} \leqslant DOD_{{\hbox{max} }}^{{i,j}} \hfill \\ K_{{ch\arg e}}^{{i,j,t}}*K_{{disch\arg e}}^{{i,j,t}}=0 \hfill \\ \end{gathered} \right.$$
  Where $K_{{ch\arg e}}^{{i,j,t}}$ and $K_{{disch\arg e}}^{{i,j,t}}$ are the battery charging and discharging status flag bits, which are 0 or 1. When $K_{{ch\arg e}}^{{i,j,t}}$ is 0, $K_{{disch\arg e}}^{{i,j,t}}$ is 1, and when $K_{{disch\arg e}}^{{i,j,t}}$ is 1, $K_{{ch\arg e}}^{{i,j,t}}$ is 0.
5. Energy trading constraints between microgrids
  18 $$\left\{ \begin{gathered} 0 \leqslant {k_1}P_{{MGk - MGi}}^{t} \leqslant P_{{MGk - MGi}}^{{\hbox{max} }} \hfill \\ 0 \leqslant {k_2}P_{{MGi - MGk}}^{t} \leqslant P_{{MGi - MGk}}^{{\hbox{max} }} \hfill \\ {k_1}*{k_2}=0 \hfill \\ \end{gathered} \right.$$
  Where ${k_1}$ and ${k_2}$ are power interaction flags, set to 0 or 1, when ${k_1}$ is 0, ${k_2}$ is 1, when ${k_2}$ is 0, ${k_1}$ is 1.

##  Sparrow search algorithm

In the sparrow search algorithm^20,21^, the sparrow population is generally divided into three subgroups: followers, discoverers, and alerts. The sparrow who leads the population to move is called the discoverer, and their position update formula is as follows:

19 $$x_{{i,j}}^{{it+1}}=\left\{ {\begin{array}{*{20}{c}} {x_{{i,j}}^{{it}}*\exp ( - \frac{i}{{\alpha *iter}})}&{R2<ST} \\ {x_{{i,j}}^{t}+Q*L}&{R2 \geqslant ST} \end{array}} \right.$$

Where *i* is the sparrow’s number, $i=1,2,3, \cdots ,n$; *j* is optimization dimension, $j=1,2,3, \cdots ,D$; $\alpha \in (0,1]$; $R2 \in [0,1]$; $ST \in [0.5,1]$; *L* is a 1×D matrix; when $R2<ST$, it is relatively safe and the discoverer will actively expand the search range to search for food; when $R2 \geqslant ST$, it indicates that the population is in a poor position and there is danger around it, so it should leave the current location.

Followers monitor the movement of the discoverer and compete for the position. When the follower fails to compete for position, they will fly to other positions, and the position update is as follows:

20 $$x_{{i,j}}^{{it+1}}=\left\{ {\begin{array}{*{20}{c}} {Q*\exp (\frac{{{x_{worst}} - x_{{i,j}}^{{it}}}}{{{i^2}}})}&{i>\frac{{{n_1}}}{2}} \\ {x_{P}^{{it+1}}+\left| {x_{{i,j}}^{{it}} - x_{P}^{{it+1}}} \right|*{A^+}*L}&{i \leqslant \frac{{{n_1}}}{2}} \end{array}} \right.$$

Where *A* is a 1×D matrix, and the values of the elements in the matrix are randomly selected from 1 or −1; ${A^+}={A^T}{(A{A^T})^{ - 1}}$; ${n_1}$ is the set number of followers; When $i>\frac{{{n_1}}}{2}$, it indicates that the follower is hungry and has not received food, and needs to go to other locations to obtain food to improve their fitness value; When $i \leqslant \frac{{{n_1}}}{2}$, it indicates that the follower has obtained food; *L* is the population size minus the number of discoverers and vigilantes. When discoverers and vigilantes account for 20% and 10% of the population, respectively, 80% of the followers will rush forward to grab food, while the remaining 20% will continue to fly to other locations to search for food.

The alert personnel are responsible for investigating and warning, informing of the danger in advance, and their proportion is 10% or 20% of the sparrow population. The updated location is as follows:

21 $$x_{{i,j}}^{{it+1}}=\left\{ {\begin{array}{*{20}{c}} {x_{{best}}^{{it}}+\beta *\left| {x_{{i,j}}^{{it}} - x_{{best}}^{{it}}} \right|}&{{f_i}>f} \\ {x_{{i,j}}^{{it}}+k*\frac{{\left| {x_{{i,j}}^{{it}} - x_{{worst}}^{{it}}} \right|}}{{{f_i} - {f_w}+\varepsilon }}}&{{f_i}={f_g}} \end{array}} \right.$$

Where *k* is a random number and $$k \in \left[ {\begin{array}{*{20}{c}} { - 1}&1 \end{array}} \right]$$; $\varepsilon$ is a constant that is generally small, used to avoid the denominator being 0;when ${f_i}>{f_g}$, sparrows are vulnerable to attacks from natural enemies; when ${f_i}={f_g}$, sparrows realize that danger is approaching other sparrows.

**Fig. 1:**
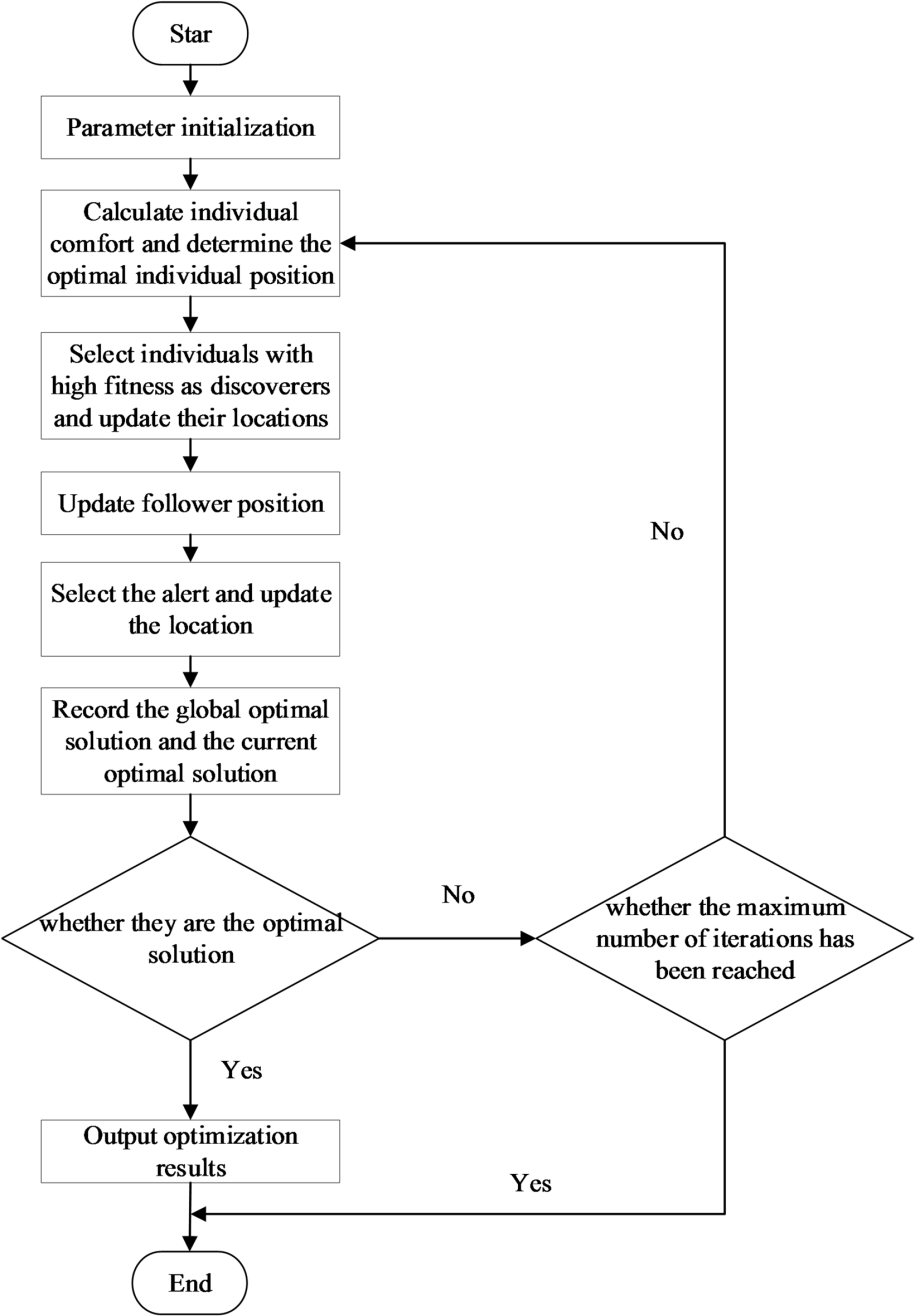
Calculation flowchart.

The flowchart of the sparrow search algorithm is shown in Fig. 1, with the following steps:

**Step 1**: Initialize the population.

First, randomly generate the initial position and velocity of the sparrow population, determine the population size, maximum iteration times, discoverers, followers, alerts, and other parameters.

**Step 2:** Calculate the fitness value.

Calculating the fitness value of each sparrow individual according to the objective function of the optimization problem, and comparing the advantages and disadvantages of the optimization space based on the fitness value.

**Step 3:** Update the position of the discoverer.

The discoverer explores the search space with a larger step size to find food, and determines the optimal location of the food source based on each experience and the current global optimal solution.

**Step 4:** Update the follower’s position.

After the discoverer finds food, the follower will consider the distance from the discoverer and their own fitness, and choose to join a group of discoverers to update their position. Followers can obtain better fitness values by having relatively smaller movement steps because they are conducting local searches around the discoverer.

**Step 5:** The alert will detect the danger and update the location.

During the iteration process, the alert group will detect the presence of danger in the surrounding area with a certain probability. If danger is detected, the alert will sound an alarm, and other individual sparrows will move to a safe area based on the alarm information and update their position.

**Step 6:** Determine the termination condition.

If the termination condition is met, output the current optimal solution; Otherwise, return to **step 2** to continue iterating.

## Case study

**Fig. 2:**
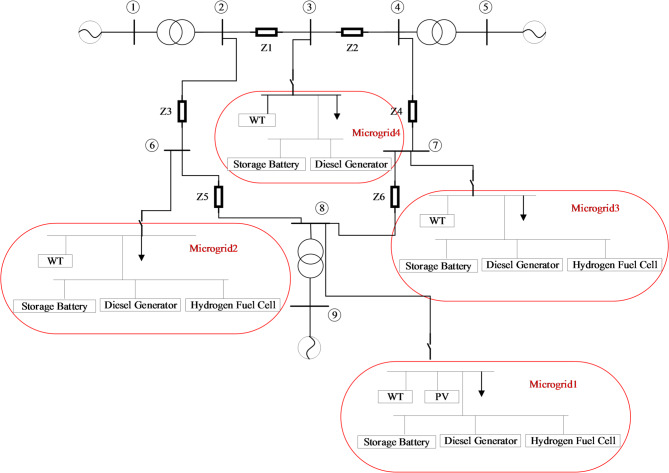
System architecture topology diagram.

To verify the feasibility of the method, four microgrids were connected to the IEEE9 system to form a microgrid cluster. The system structure is shown in Fig. 2, where the parameters of distributed power sources are shown in Table 1, and the time of use electricity price of the power grid is shown in Table 2.

**Table 1:** Distributed power generation parameters.

Name	Lower limit of output (kW)	Upper limit of output (kW)
WT	0	80
PV	0	60
Storage battery	− 50	50
Diesel generator	0	40
Hydrogen fuel cell	− 50	50

**Table 2:** Time-of-use price.

Name	Peak (Yuan/kWh)	Shoulder (Yuan/kWh)	Off-peak (Yuan/kWh)
Selling electricity	0.65	0.38	0.13
Purchasing electricity	0.83	0.49	0.27

(1) Selecting typical winter days as simulation objects for the system, the sparrow search algorithm is used to optimize the economic scheduling of the microgrid cluster. The population size is 500. The number of discoverers is 648. The maximum number of iterations is 150. The warning value is 0.6. The load data of each microgrid is shown in Fig. 3. The local weather conditions are shown in Figs. 4, 5, 6. The comparison curves of the microgrid cluster are shown in Figs. 7, 8, 9, 10, 11, 12, 13, 14, 15, 16, 17, 18 based on this method and particle swarm algorithm.

**Fig. 3:**
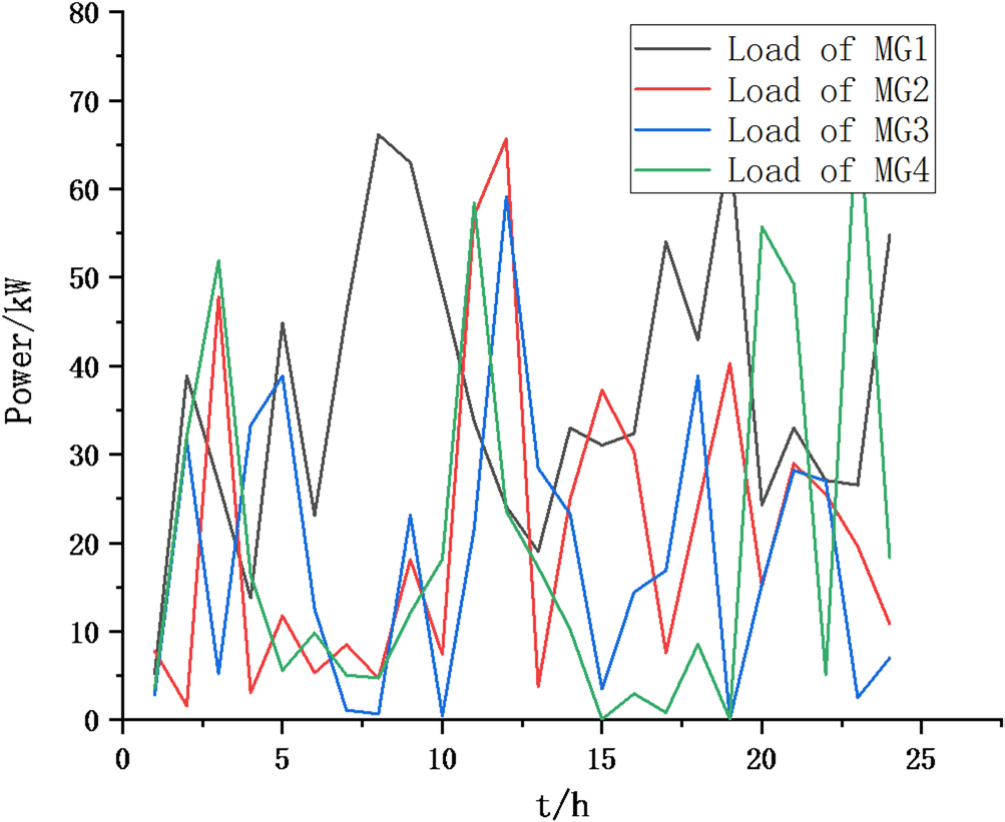
The load data of each microgrid.

**Fig. 4:**
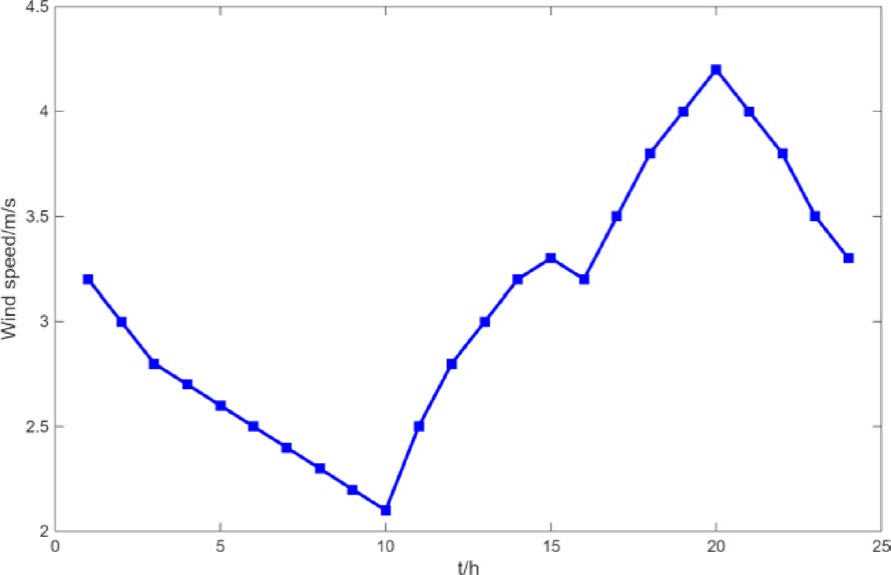
The curve of wind speed.

**Fig. 5:**
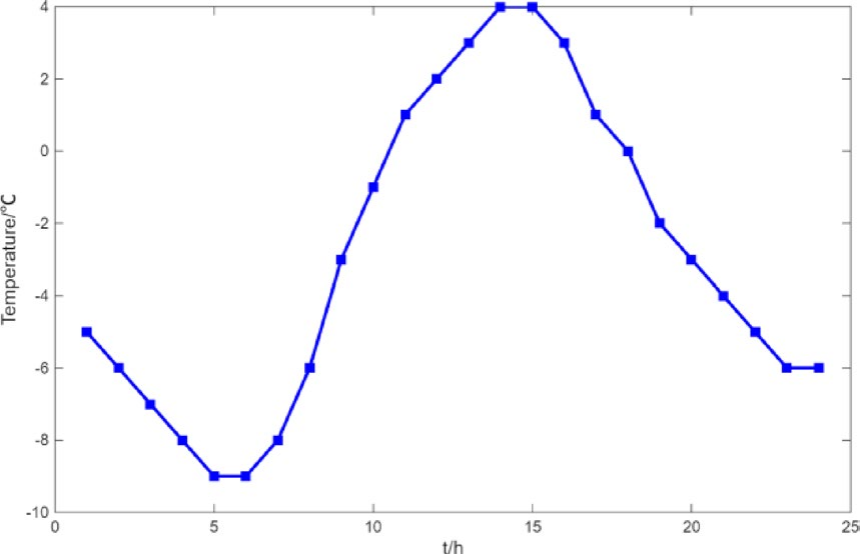
The curve of temperature.

**Fig. 6:**
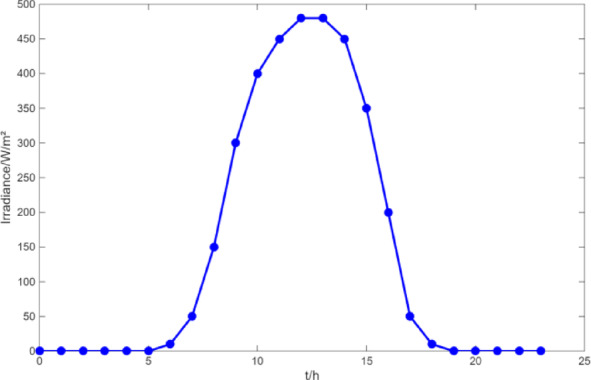
The curve of irradiance.

**Fig. 7:**
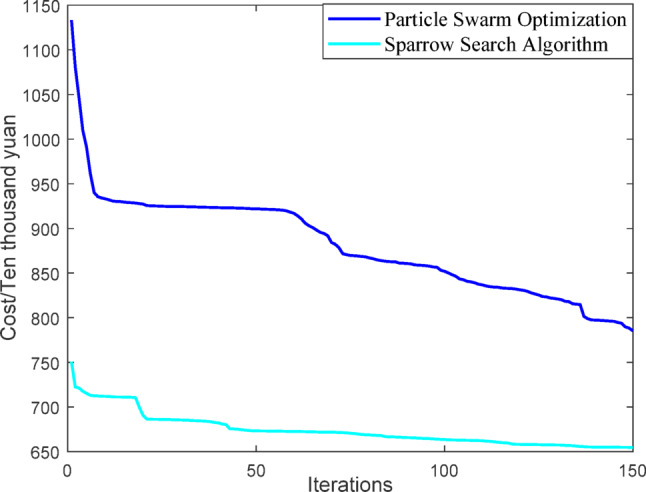
Iterative curves of different algorithms.

**Fig. 8:**
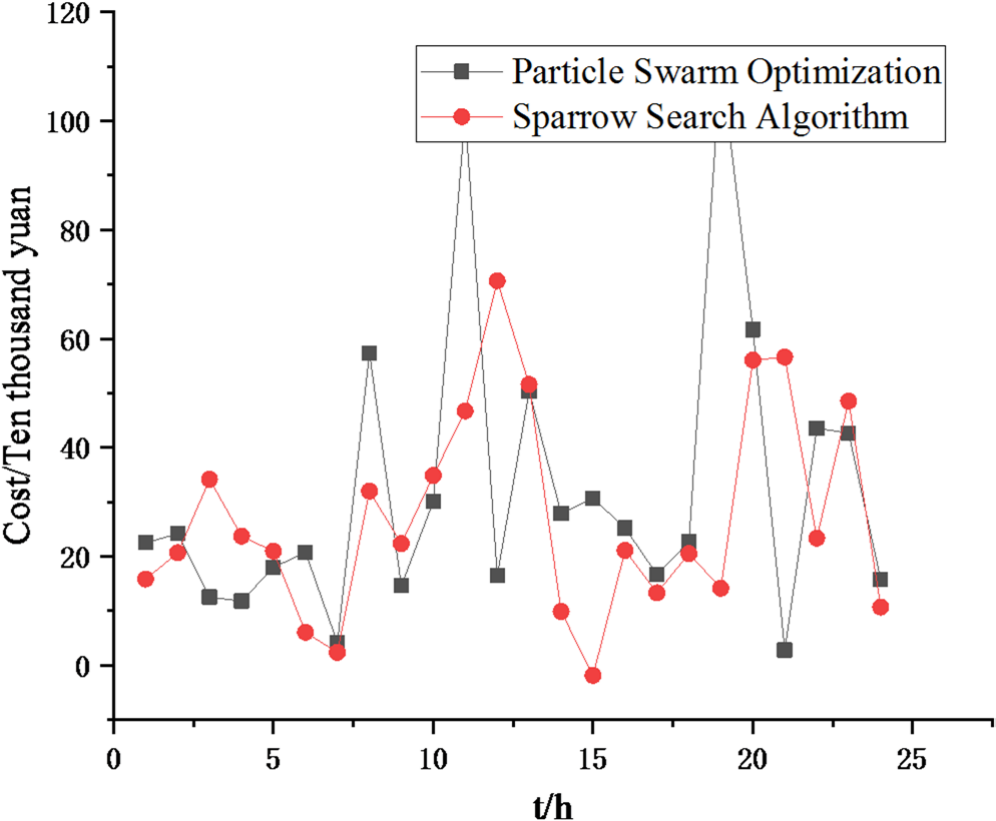
Comparison curve of different algorithm costs.

In Figs. 7 and 8, it can be seen that the sparrow search algorithm ultimately finds a better solution than the particle swarm algorithm, meaning that the cost of the final iteration is lower. And the final iteration cost of the particle swarm optimization algorithm is approximately 1.2 times that of the sparrow search algorithm. Thus, the sparrow search algorithm is better.

**Fig. 9:**
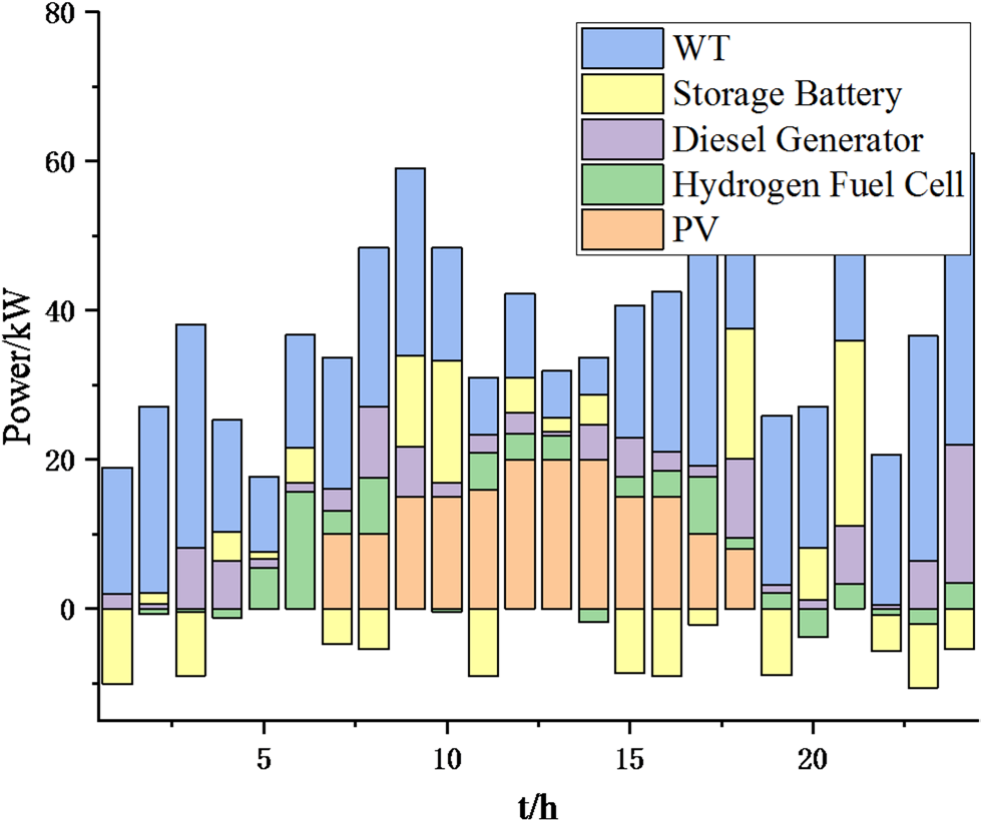
Operation of microgrid 1 based on particle swarm optimization algorithm.

**Fig. 10:**
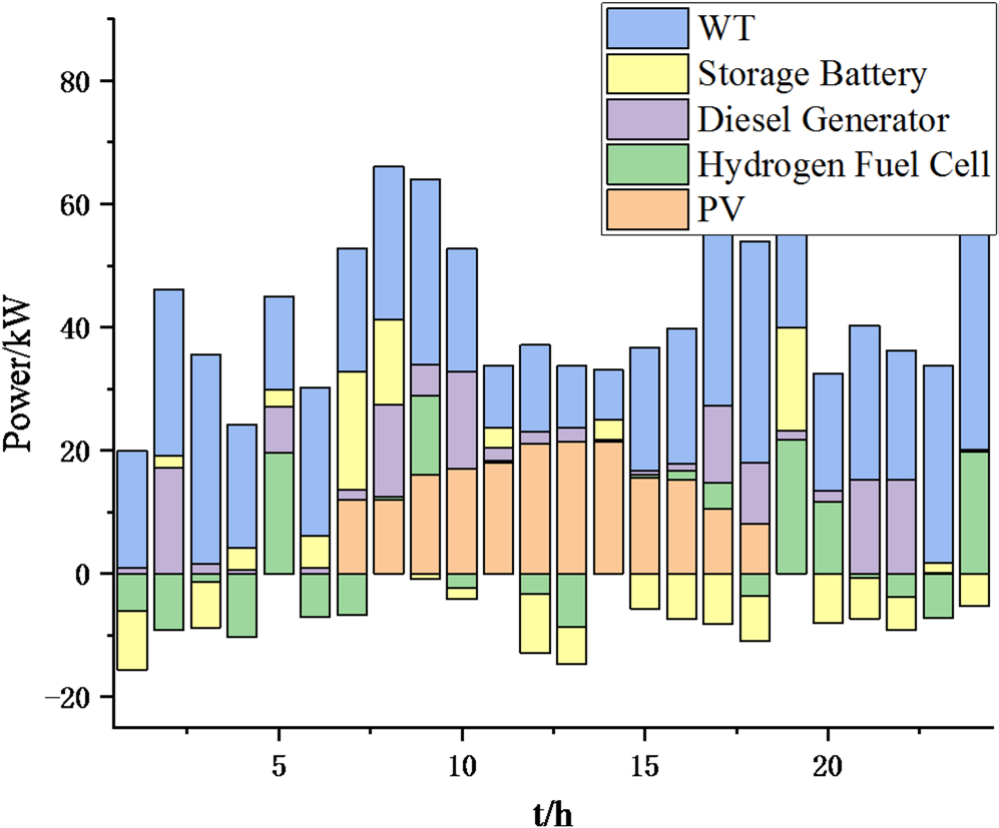
Operation of microgrid 1 based on sparrow search algorithm.

**Fig. 11:**
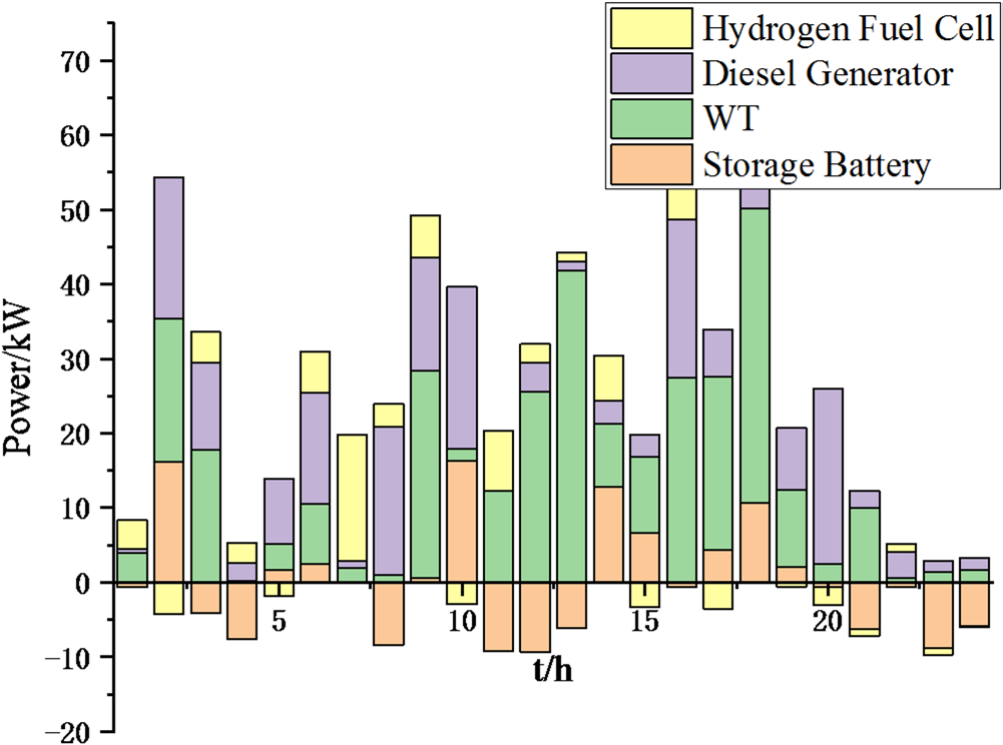
Operation of microgrid 2 based on particle swarm optimization algorithm.

**Fig. 12:**
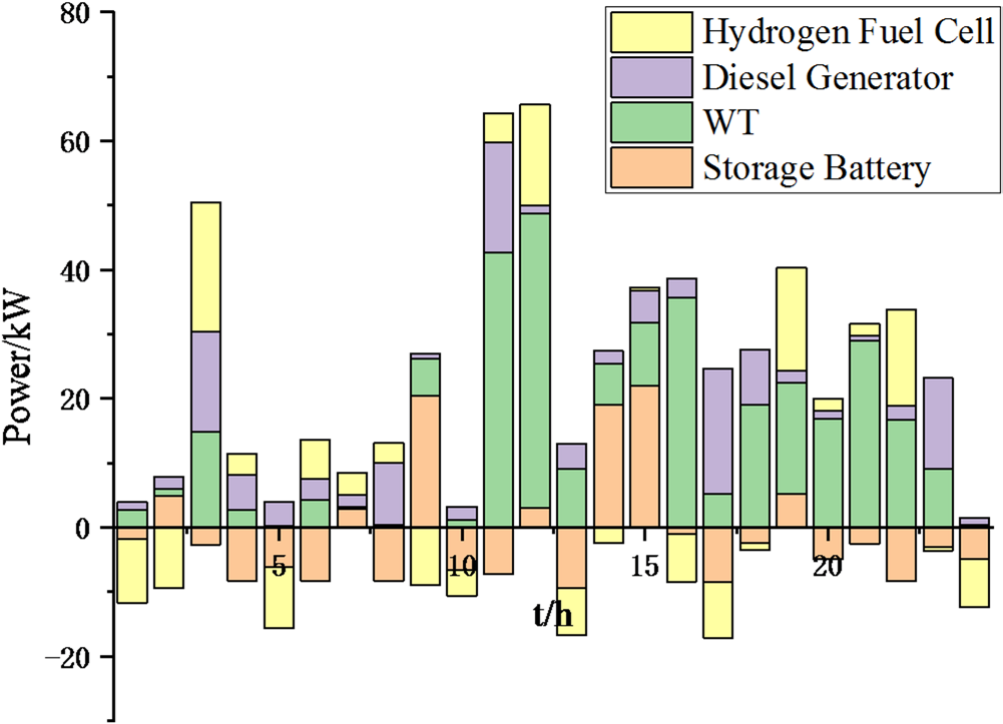
Operation of microgrid 2 based on sparrow search algorithm.

**Fig. 13:**
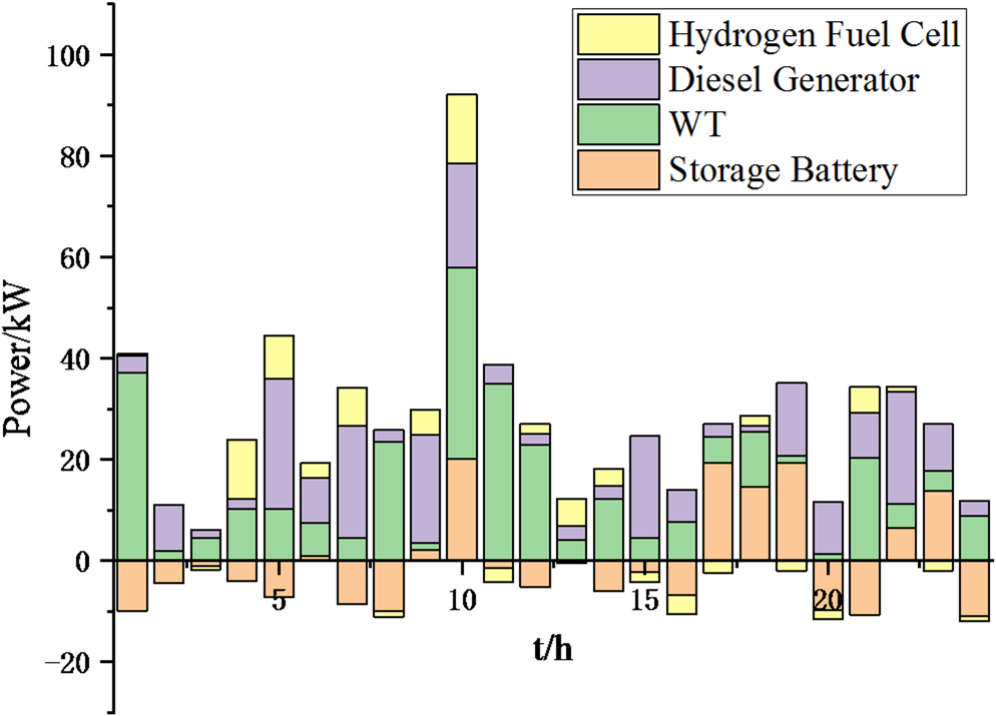
Operation of microgrid 3 based on particle swarm optimization algorithm.

**Fig. 14:**
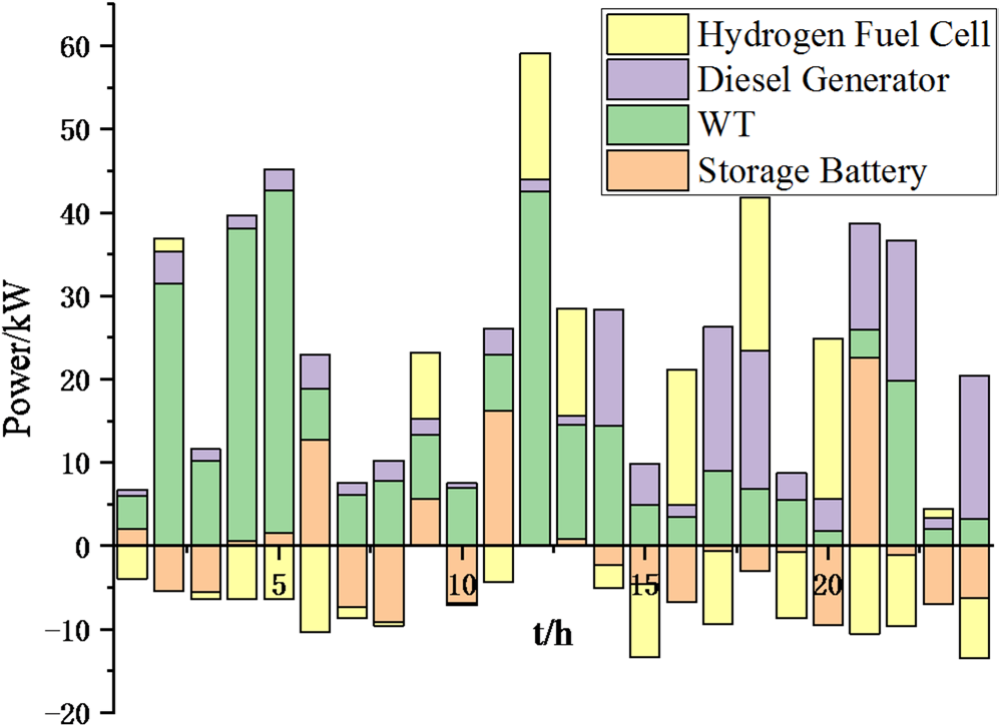
Operation of microgrid 3 based on sparrow search algorithm.

**Fig. 15:**
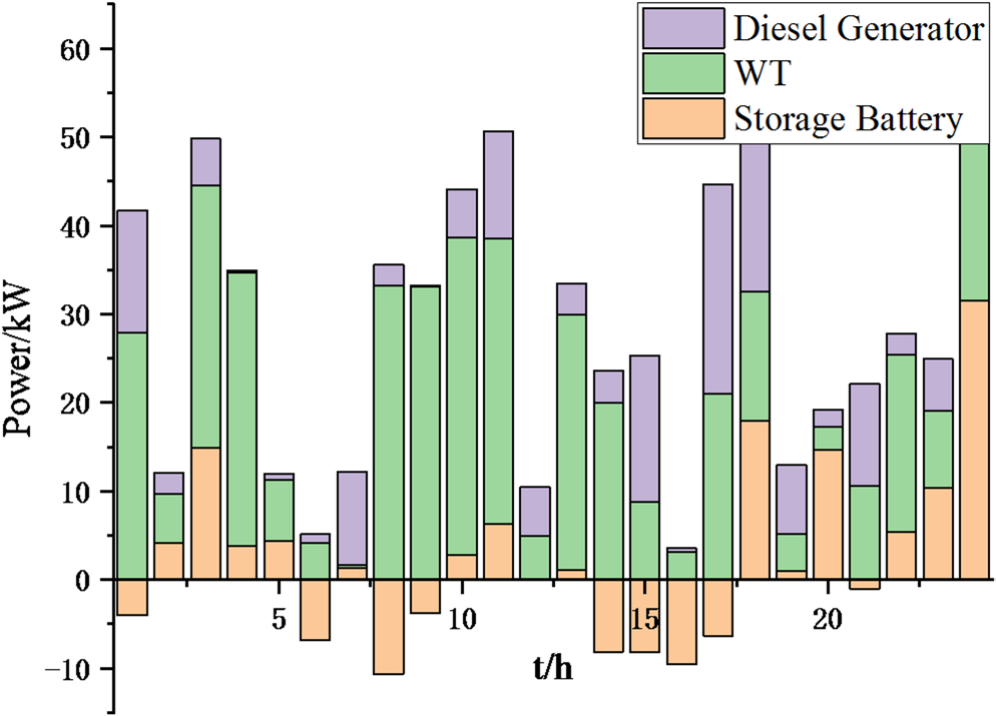
Operation of microgrid 4 based on particle swarm optimization algorithm.

**Fig. 16:**
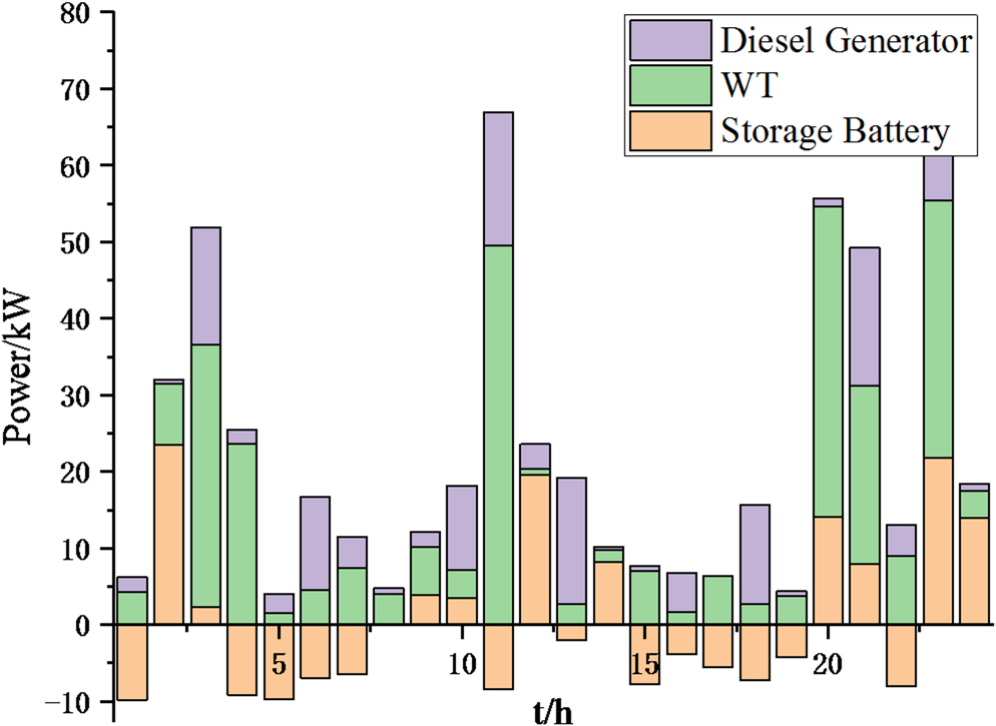
Operation of microgrid 4 based on sparrow search algorithm.

From Figs. 9, 10, 11, 12, 13, 14, 15 and 16, it can be seen that compared to the particle swarm optimization algorithm, renewable energy can be utilized more fully based on the sparrow search optimization algorithm, such as WT and PV, which can achieve maximum utilization within the predicted range. The charging and discharging strategies for batteries and hydrogen fuel cells have become more reasonable, enabling charging at low electricity prices and discharging at high prices. Diesel generators are more economical to use as backup power sources, reducing operating time and thus lowering air pollution.

**Fig. 17:**
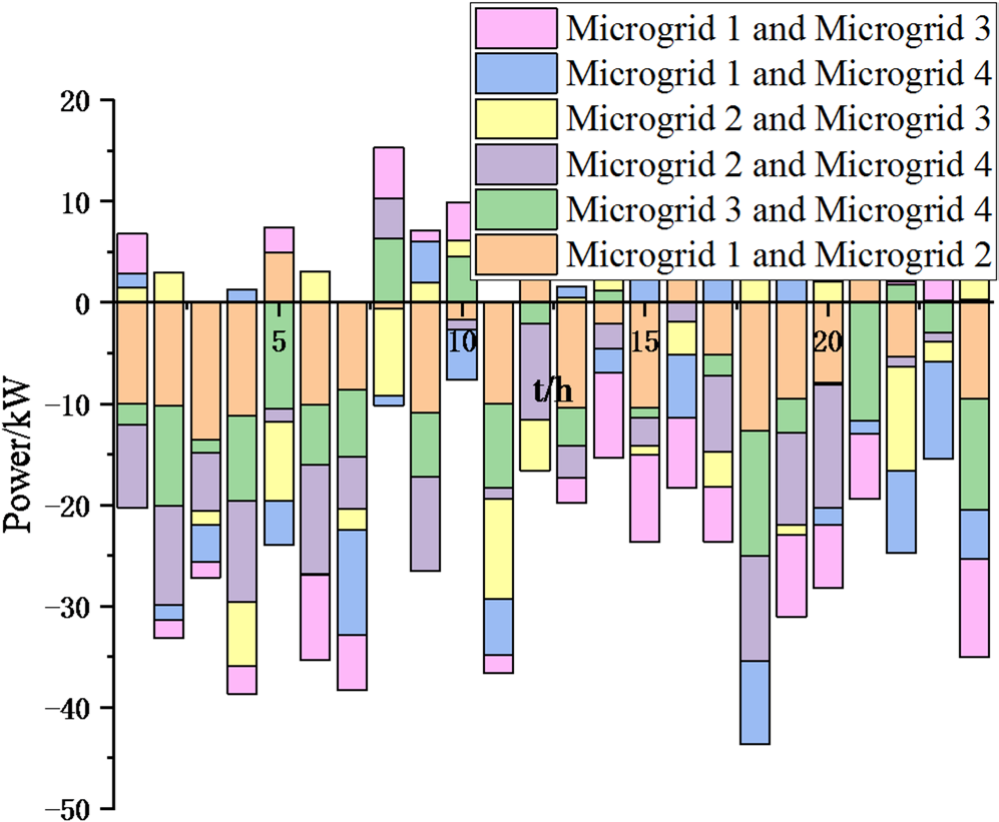
Electricity exchange between microgrids based on particle swarm optimization algorithm.

**Fig. 18:**
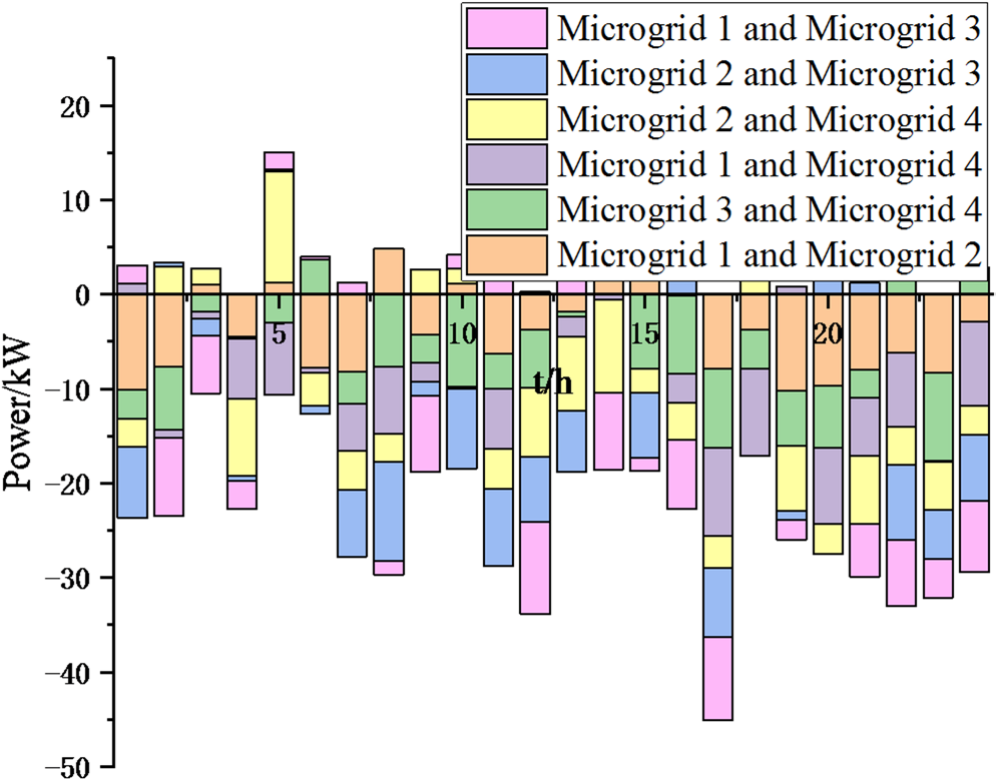
Electricity exchange between microgrids based on sparrow search algorithm.

In Figs. 17 and 18, it can be seen that compared to the particle swarm optimization algorithm, after adopting the sparrow search optimization algorithm, the exchange of electrical energy between microgrids is more frequent and reasonable, which can greatly reduce energy waste, achieve resource complementarity, reduce unnecessary interaction costs, thereby reducing cost waste and lowering system operation costs. Thus, this method is feasible.

(2) To further verify the feasibility of the method, a certain day in summer is selected as the simulation object. The parameter settings of the optimization algorithm are consistent with the previous ones. The load data of each microgrid is shown in Fig. 19. The local weather conditions are shown in Figs. 20, 21, 22. The comparison curves of microgrid clusters are shown in Figs. 23, 24, 25, 26, 27, 28, 29, 30, 31, 32, 33, 34.

**Fig. 19:**
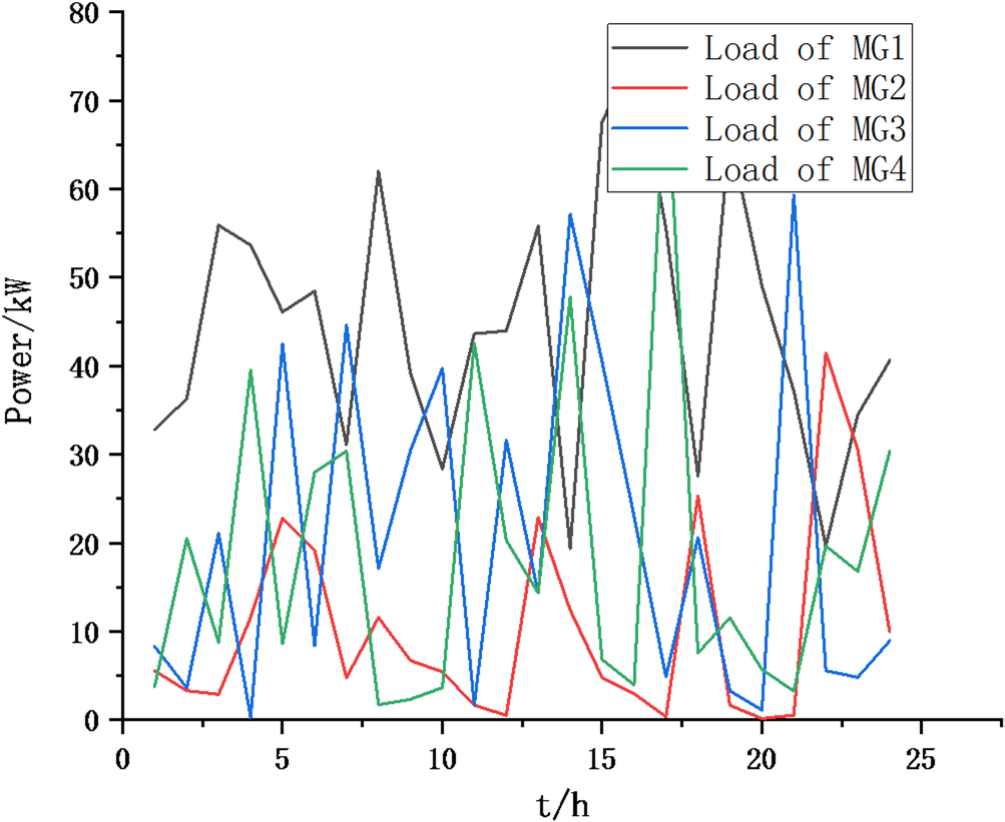
The load data of each microgrid.

**Fig. 20:**
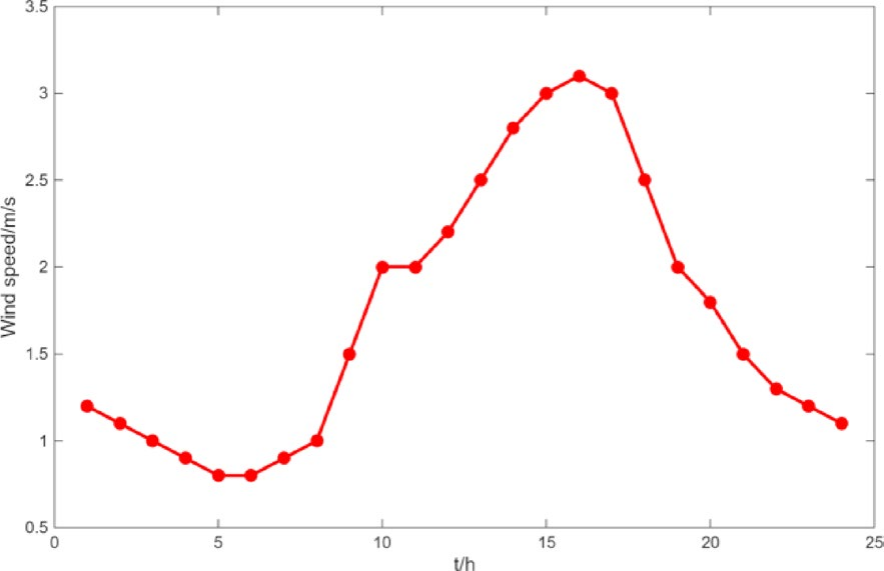
The curve of wind speed.

**Fig. 21:**
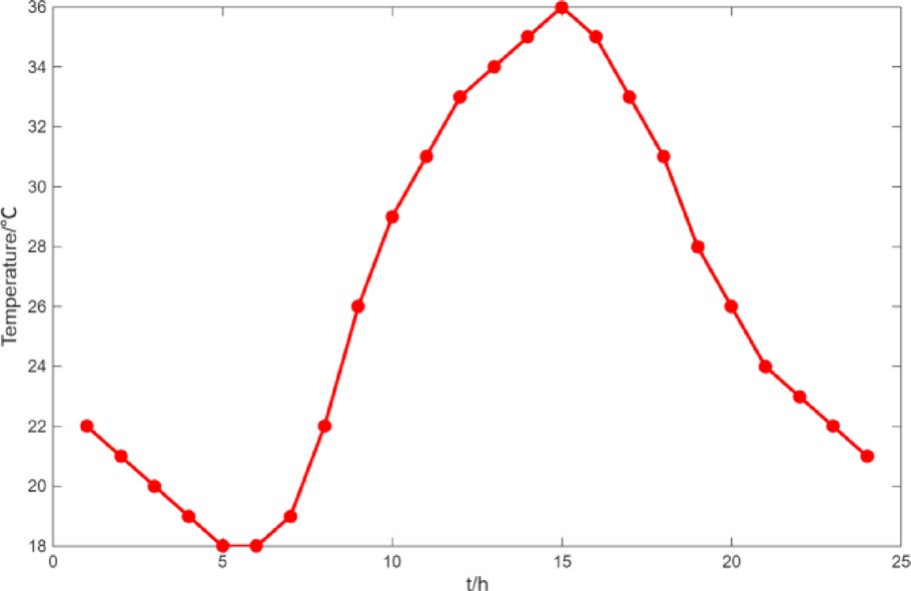
The curve of temperature.

**Fig. 22:**
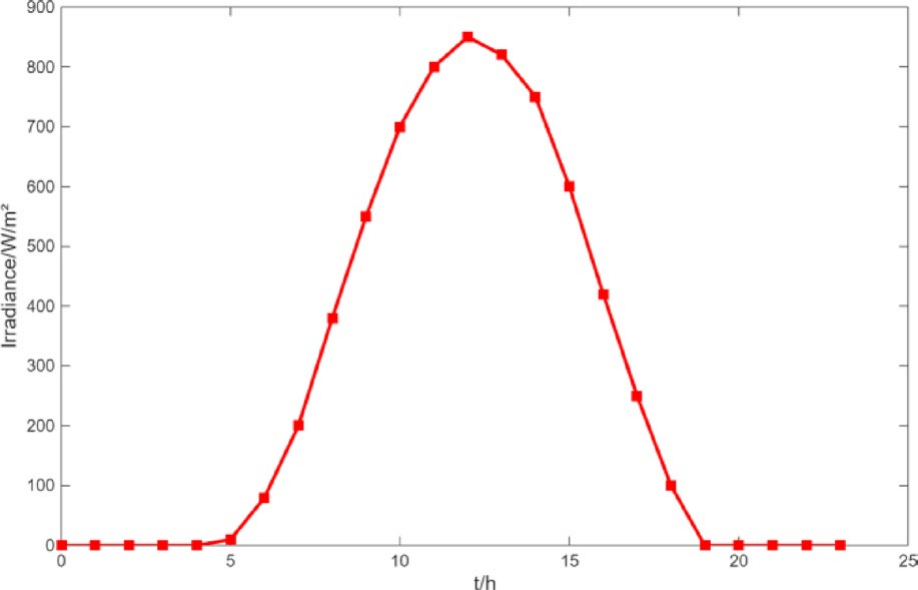
The curve of irradiance.

**Fig. 23:**
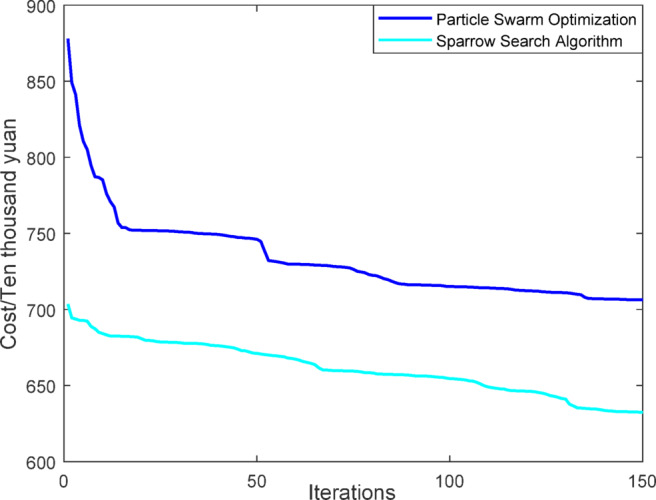
Iterative curves of different algorithms.

**Fig. 24:**
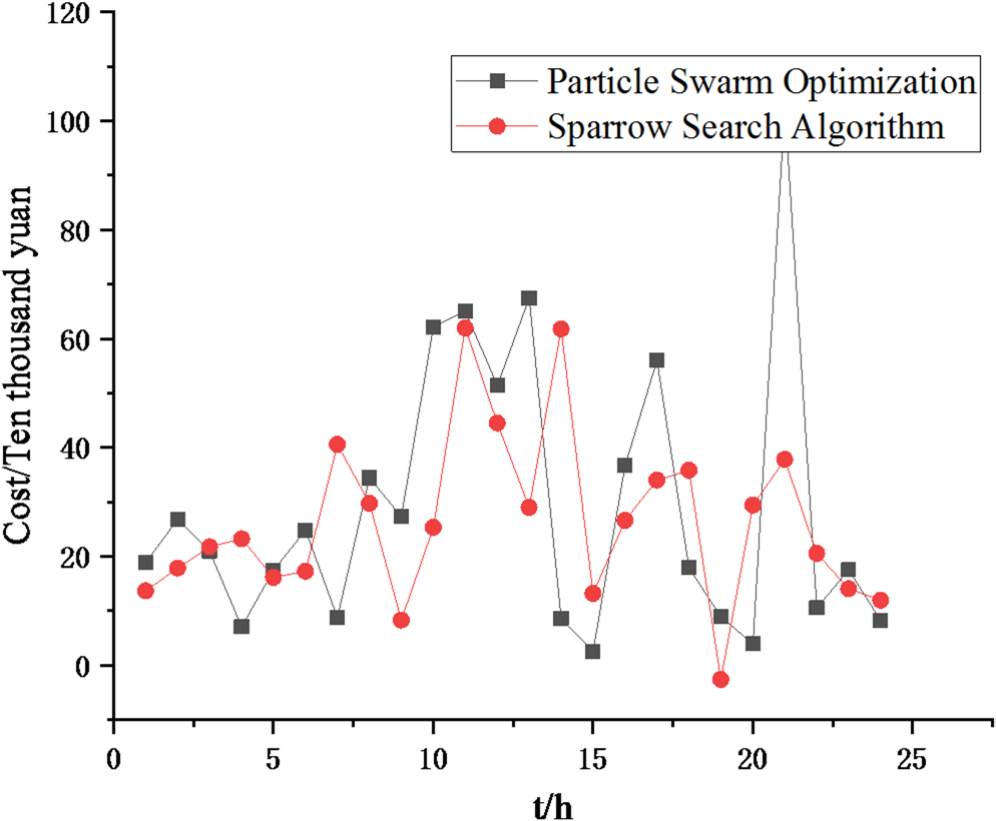
Comparison curve of different algorithm costs.

**Fig. 25:**
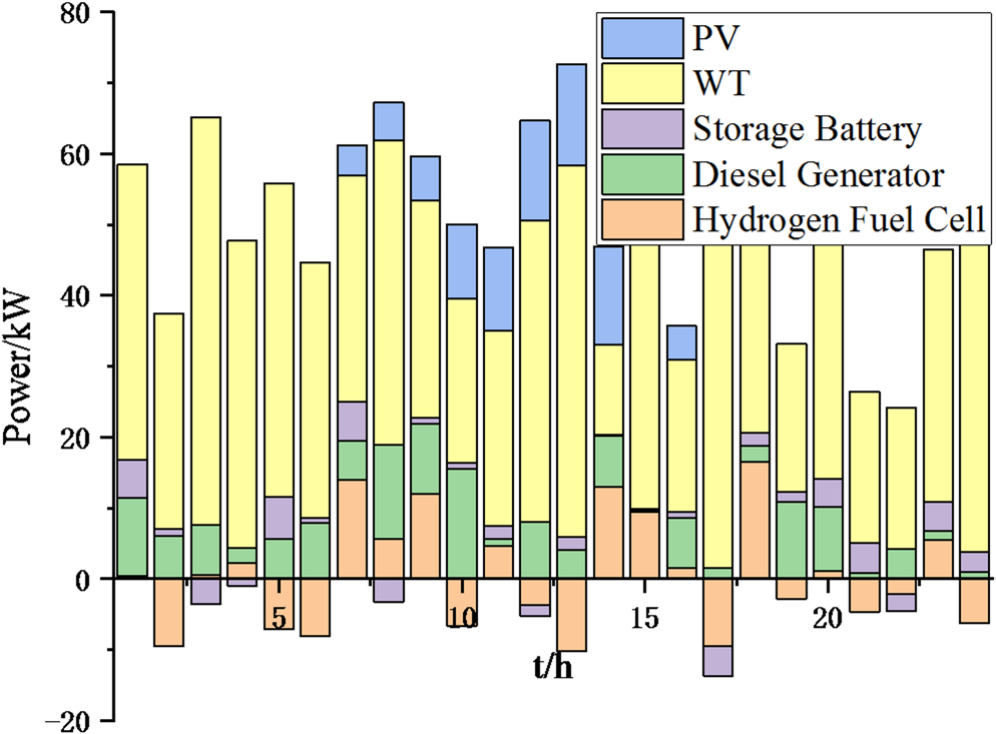
Operation of microgrid 1 based on particle swarm optimization algorithm.

**Fig. 26:**
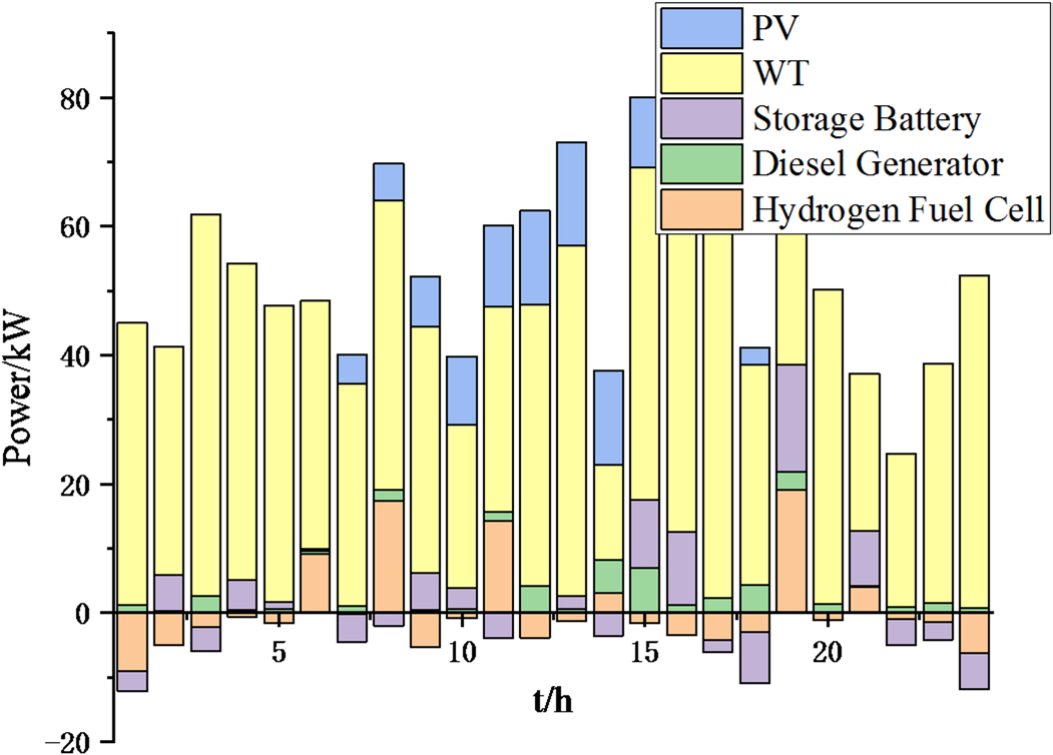
Operation of microgrid 1 based on sparrow search algorithm.

**Fig. 27:**
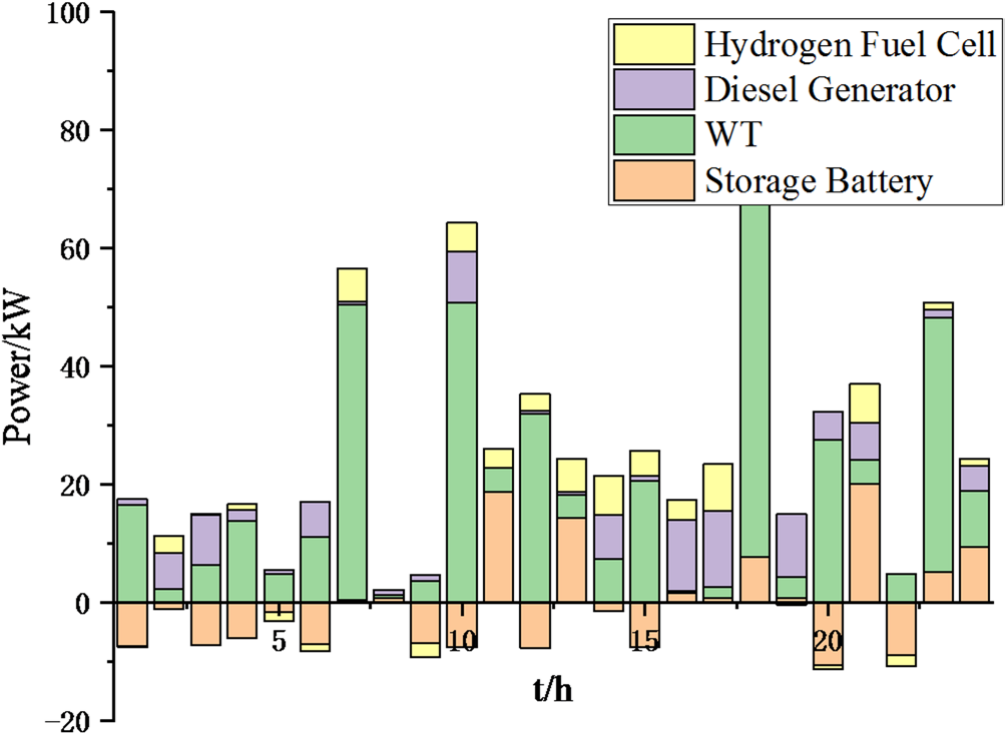
Operation of microgrid 2 based on particle swarm optimization algorithm.

**Fig. 28:**
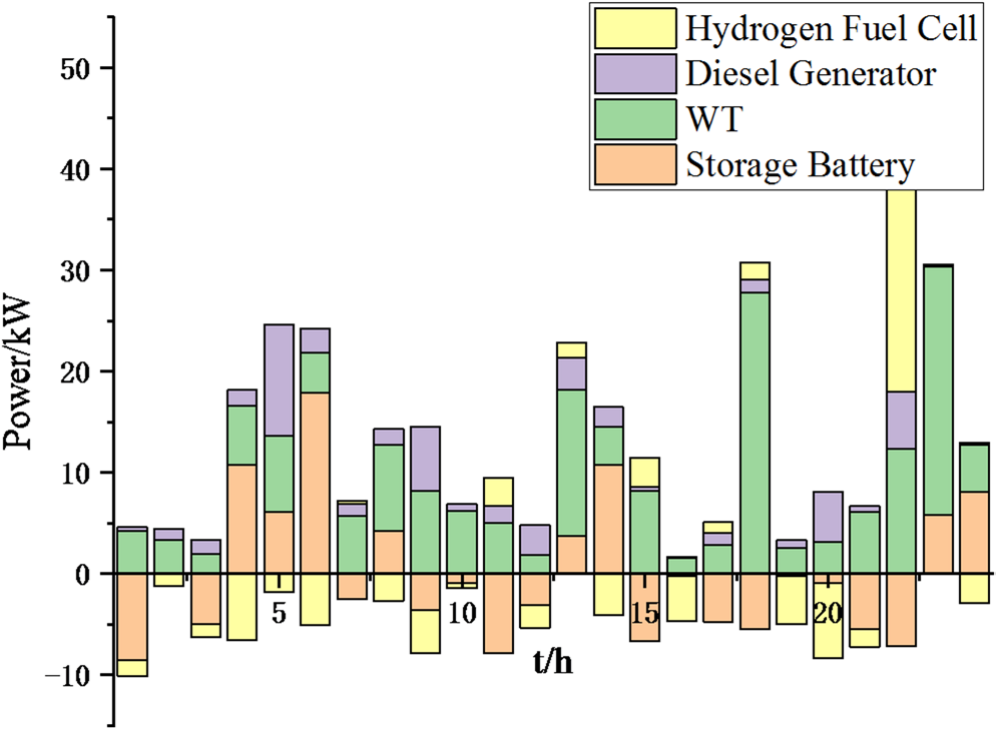
Operation of microgrid 2 based on sparrow search algorithm.

**Fig. 29:**
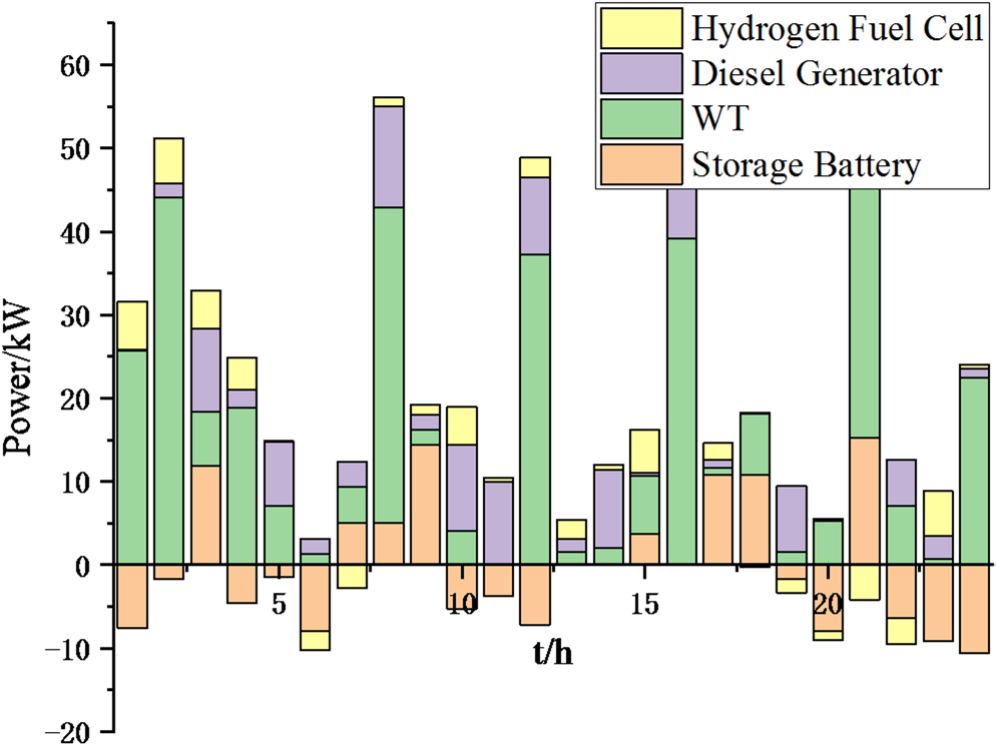
Operation of microgrid 3 based on particle swarm optimization algorithm.

**Fig. 30:**
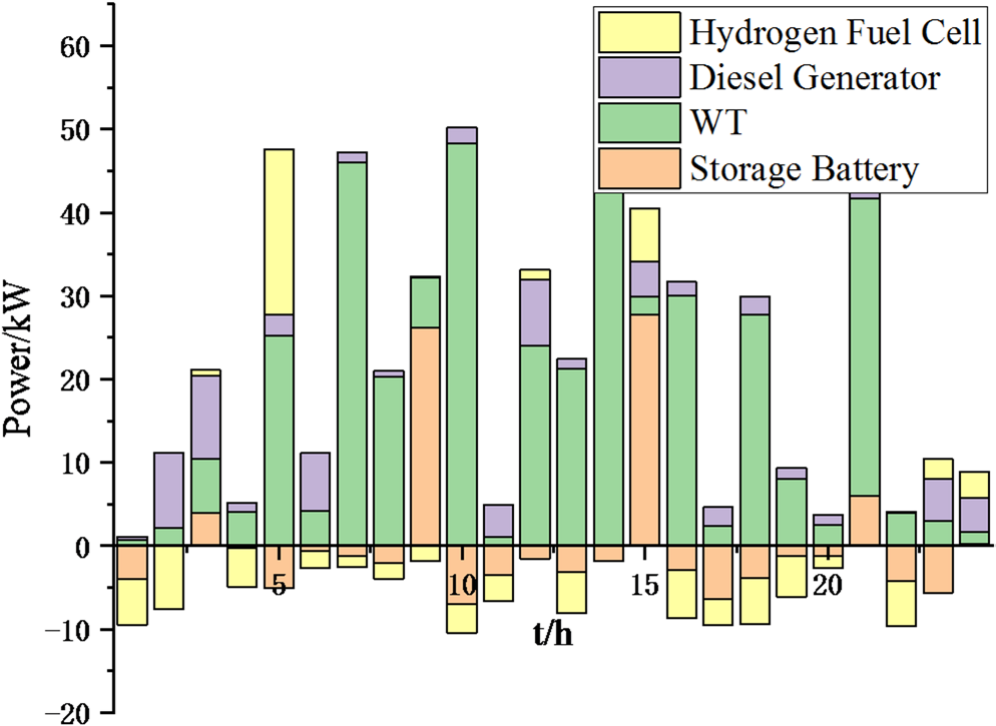
Operation of microgrid 3 based on sparrow search algorithm.

**Fig. 31:**
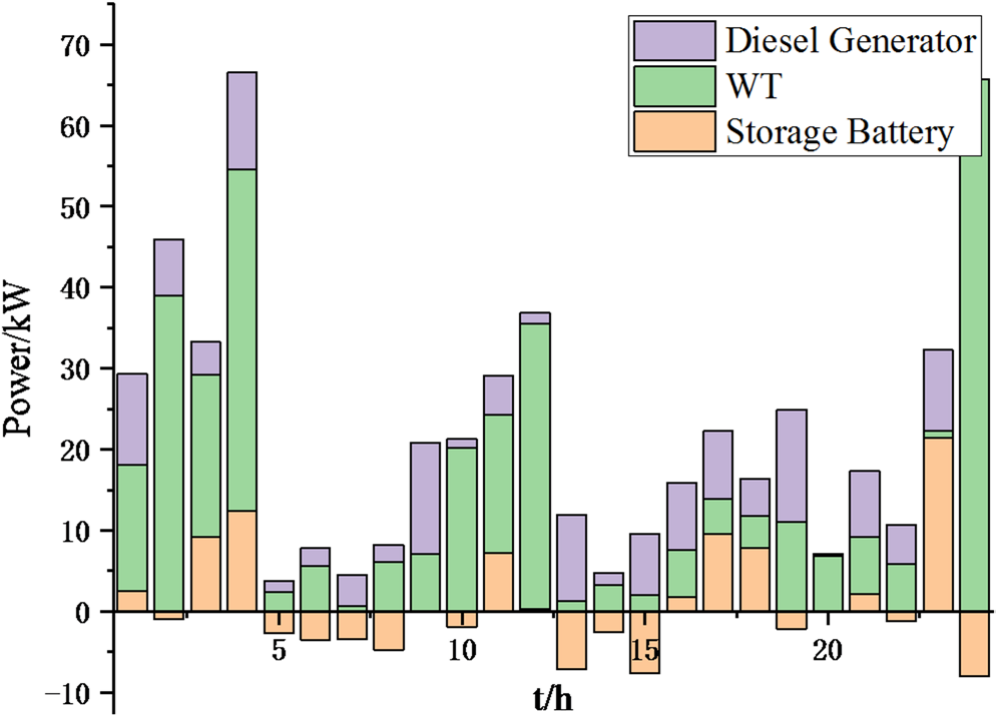
Operation of microgrid 4 based on particle swarm optimization algorithm.

**Fig. 32:**
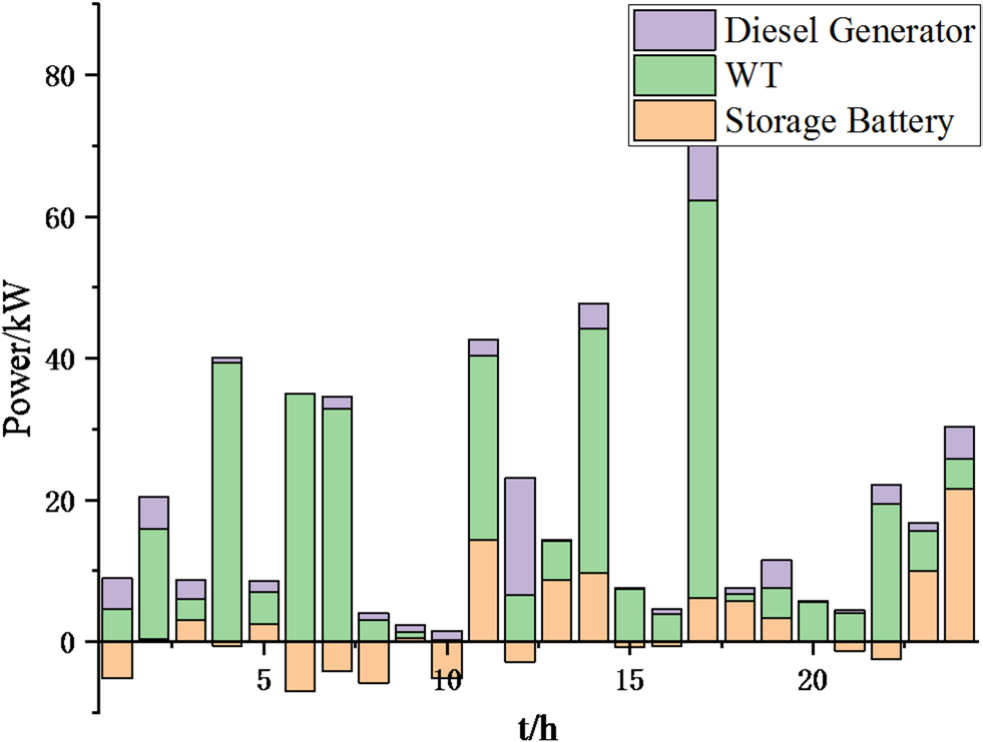
Operation of microgrid 4 based on sparrow search algorithm.

**Fig. 33:**
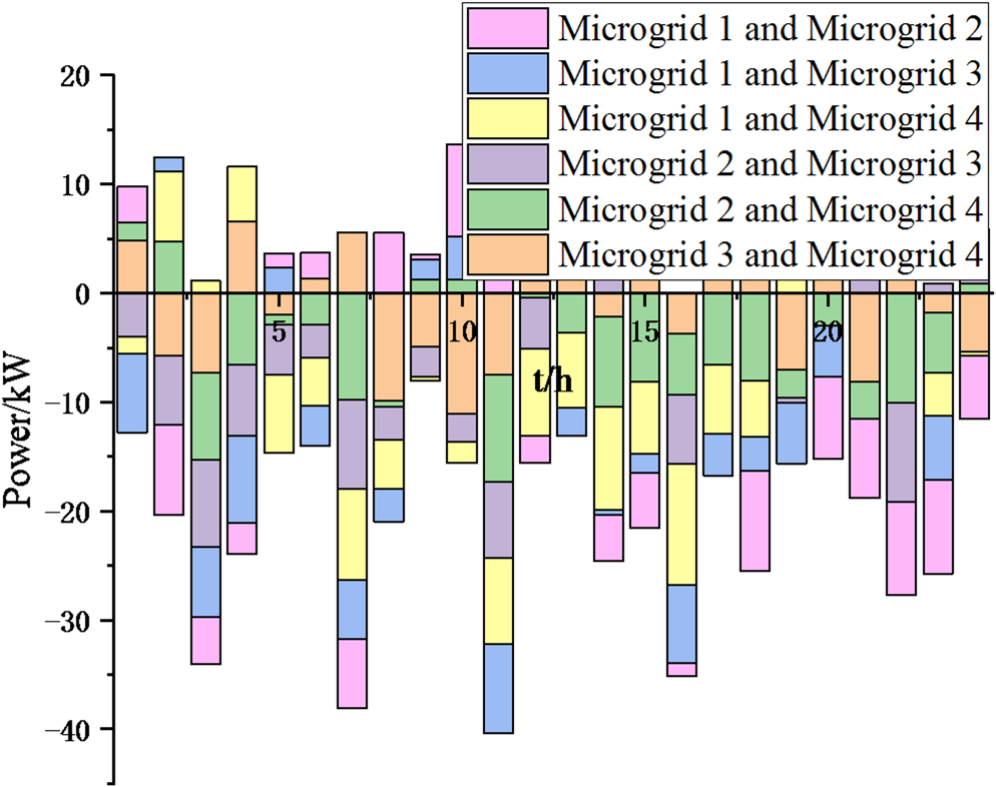
Electricity exchange between microgrids based on particle swarm optimization algorithm.

**Fig. 34:**
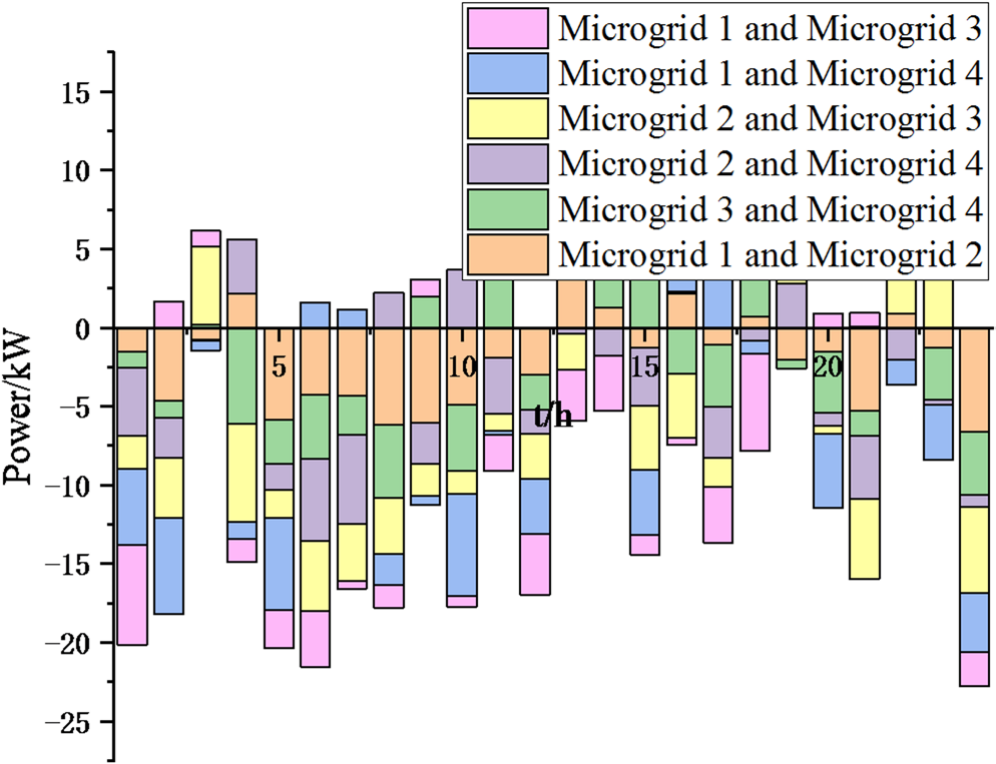
Electricity exchange between microgrids based on sparrow search algorithm.

From Figs. 23, 24, 25, 26, 27, 28, 29, 30, 31, 32, 33 and 34, it can be seen that the optimization effect of this method is better than that of the particle swarm optimization algorithm, which can greatly reduce the cost of system operation and achieve significant economic benefits. The DG scheduling strategy is more reasonable and can maximize the utilization of renewable energy. The power exchange scheme between microgrids is more optimal and achieves resource complementarity. This further proves the feasibility of the method.

## Conclusion

A microgrid cluster is a system composed of multiple microgrids, each containing DGs such as PV, WT, energy storage devices, etc. Electrical energy can be exchanged with the main grid or flow between internal microgrids. Thus, the microgrid cluster plays an important role in electricity market transactions. For the microgrid cluster, economic optimization operation is the key to demonstrating its superiority. To achieve efficient energy utilization and cost-minimizing operation of the microgrid cluster, this paper studies DG models such as WT, PV, diesel generator, Energy storage battery, and hydrogen fuel cell. Taking the total maintenance costs, operating costs, and transaction costs as the objective function, and combined with time-of-use electricity prices, a microgrid cluster optimization model is proposed. To obtain the optimal solution of the optimization model, the sparrow search algorithm is used to solve the optimization model, and the feasibility and correctness of the proposed method are verified through simulation of the improved IEEE 9-node system. This method considers the time-of-use electricity price of the power grid in the process of achieving economic optimization, aiming to achieve the overall optimization goal of the system by realizing the economic efficiency of different periods. Thus, this method can provide theoretical guidance in the economic optimization scheduling of microgrid clusters.

## Data Availability

The data that support the findings of this study are available from the authors but restrictions apply to the availability of these data, which were used under license for the current study, and so are not publicly available. Data are however available from the authors upon reasonable request.

## Parameters

$P^{\prime}_{r}$: Rated power of WT (kW)
$v^{\prime}_{r}$: Rated wind speed of the WT (m/s)
$v^{\prime}_{ci}$, $v^{\prime}_{co}$: Cut-in wind speed and cut-out wind speed of the WT (m/s)
$R^{\prime}_{pv}$: Output power of PV under standard testing conditions (kW)
$$q^{\prime}_{pv}$$: Derating coefficient
$I^{\prime}_{STC}$: Sunlight intensity under standard testing conditions (kW/m^2^)
$$\alpha^{\prime}_{p}$$: Temperature coefficient
$T^{\prime}_{stc}$: Standard operating temperature for photovoltaic array components (°)
$\eta^{ + }$, $\eta^{ - }$: Charging and discharging efficiency.
$$\eta_{xl}$$: Overall efficiency
$\dot{m}_{f}$: Fuel mass flow rate (kg/h)
$Q_{LHV}$: Low calorific value of the fuel (MJ/kg)
$P_{e,ele}^{t}$: Working power during electrolysis (kW)
$\eta_{ele}^{{H_{2} }}$: Electric to hydrogen conversion efficiency
$\eta_{ele}^{{h_{e} }}$: Electric to heat conversion efficiency
$\eta_{fc}^{e}$: Hydrogen to electricity efficiency of the fuel cell at time *t*
$\eta_{fc}^{he}$: Hydrogen to heat efficiency of the fuel cell at time *t*
$P_{{H_{2} }}^{cha}$, $P_{{H_{2} }}^{dis}$: Hydrogen power injected and output of the hydrogen storage tank (kW)
$\eta_{cha}$, $\eta_{dis}$: Efficiency of hydrogen storage tank charging and discharging
*N*: The number of microgrids
$P_{{{\mathrm{DG}}j}}$: The output power of DG* j* at time *t* (kW)
$N_{DE}$: The number of DGs
*T*: Optimizing cycle
$\alpha_{j}$, $\beta_{j}$, $\gamma_{j}$: The cost coefficient of DG *j*
$a_{m,j}$: The characteristic coefficient of the operation and maintenance cost of the DG *j*
$C_{ch4}$: The price per cubic meter of natural gas (Yuan/m^3^)
$N_{FC}$: Number of energy storage batteries
$K_{DE}$: The start-up cost of the DG (Yuan)
$n_{DE}^{j}$: The number of times the DG *j* is turned on
$N_{el}$: The number of hydrogen fuel cells
$P_{DG}^{i,j,\min }$: The lower limit of output power generation of the DG *j* in microgrid *i* (kW)
$P_{DG}^{i,j,\max }$: The upper limit of output power generation of the DG *j* in microgrid *i* (kW)
$P_{i,genj,down}^{\max }$: The maximum output reduction rate of the generator *j* in microgrid *i* at time *t* (kW)
$P_{i,genj,up}^{\max }$: The maximum output increase rate of the generator *j* in microgrid *i* at time *t* (kW)
$E_{{_{\min } }}^{i,j}$: The minimum amount of electricity that the battery *j* can store in microgrid *i* (kWh)
$E_{\max }^{i,j}$: The maximum amount of electricity that the battery *j* can store in microgrid *i* (kWh)
$P_{ch\arg e}^{i,j,\max }$: The maximum charging capacity of the battery *j* in microgrid *i* (kW)
$P_{disch\arg e}^{i,j,\max }$: The maximum discharge capacity of battery *j* in microgrid *i* (kW)
$SOC_{\min }^{i,j}$: The minimum remaining power of battery* j* in microgrid *i*
$SOC_{\max }^{i,j}$: The maximum remaining power of battery *j* in microgrid *i*
$DOD_{\min }^{i,j}$: The minimum discharge depth of battery *j* in microgrid *i*
$DOD_{\max }^{i,j}$: The maximum discharge depth of battery *j* in microgrid *i*
$P_{MGk - MGi}^{\max }$: The maximum amount of electricity purchased by microgrid *i* from microgrid *k* (kW)
$P_{MGi - MGk}^{\max }$: The maximum amount of electricity sold from microgrid *i* to microgrid *k* (kW)
*n*: The number of sparrows
*D*: The total dimensionality
*it*: The current iteration count
$iter$: The maximum number of iterations set for the algorithm
$Q$: A normally distributed random number
$\alpha$: A random number randomly selected from the range
*R*2: The warning value
*ST*: The safety value
$x_{worst}$: The position with the worst fitness value in the algorithm search range
$x_{P}^{it + 1}$: The optimal location that has been identified by the current discoverer
$x_{best}^{t}$: The current global optimal position
*β*: A standard normal distribution random number
$f_{i}$: The fitness value of the current sparrow individual
$f_{g}$: The fitness value of the global optimal position
$f_{w}$: The fitness value of the global worst position

## Variables

$P_{bess} (t)$: Charging and discharging power of the energy storage battery at time *t* (kW)
$P^{\prime}_{WT} (t)$: Output power of WT at the time *t* (kW)
$I^{\prime}_{T} (t)$: Actual sunlight intensity at time *t* (kW/m^2^)
$T^{\prime}_{c} (t)$: Actual operating temperature of the photovoltaic array components at time *t* (°)
$P^{\prime}_{pv} (t)$: Output power of PV at the time *t* (kW)
$SOC(t)$: Remaining power of the energy storage battery at time *t*
$P_{{{\mathrm{gen}}}} (t)$: Output power of the diesel generator at the time *t* (kW)
$P_{{H_{2} ,ele}}^{t}$: Hydrogen production power of the electrolytic cell at time *t* (kW)
$P_{{H_{2} ,fc}}^{t}$: Working power of the fuel cell at time *t* (kW)
$P_{{h_{e} ,ele}}^{t}$: Generated waste heat power at time* t* (kW)
$P_{e,fc}^{t}$: Power generation of the fuel cell at time *t* (kW)
$P_{{h_{e} ,fc}}^{t}$: Residual heat power generated by hydrogen combustion in the fuel cell at time *t* (kW)
$E_{ht}^{t}$: Remaining amount of hydrogen storage tank at time *t* (kWh)
$u_{DE}^{j,t}$: The switch state of DG* j* at time *t*
$P_{DG}^{j,t}$: The output power of DG* j* at time* t* (kW)
$P_{FC}^{j,t}$: The output power of energy storage battery *j* at time *t* (kW)
$f_{DE}^{i}$: The power generation cost of DGs in microgrid *i*, (Yuan)
$f_{FC}^{i}$: The power generation cost of energy storage battery in microgrid *i* (Yuan)
$f_{m}^{i}$: The operation and maintenance cost of each DG in microgrid *i* (Yuan)
$f_{s}^{i}$: The start stop cost of controllable DGs in microgrid *i* (Yuan)
$f_{buy}^{i}$: The cost of electricity from microgrid *i* to the power grid and microgrid* j* (Yuan)
$f_{sell}^{i}$: The cost of selling electricity from microgrid *i* to the power grid and microgrid *j* (Yuan)
$f_{{{\mathrm{el}}}}^{i}$: The power generation cost of hydrogen fuel cells in microgrid *i* (Yuan)
$q_{grid - MGi}^{t}$: The unit purchase price of electricity from microgrid* i* to the power system at time *t* (Yuan/kWh)
$P_{grid - MGi}^{t}$: The amount of electricity purchased by microgrid *i* from the power grid at the time *t* (kWh)
$q_{MGk - MGi}^{t}$: The unit purchase price of electricity from microgrid *i* to microgrid *k* at time* t* (Yuan/kWh)
$P_{MGk - MGi}^{t}$: The amount of electricity purchased by microgrid *i* from microgrid *k* at the time *t* (kWh)
$q_{MGi - grid}^{t}$: The unit selling price of electricity from microgrid* i* to the power grid at time *t* (Yuan/kWh)
$P_{MGi - grid}^{t}$: The amount of electricity sold from microgrid *i* to the power grid at the time *t* (kWh)
$q_{MGi - MGk}^{t}$: The unit selling price of electricity from microgrid *i* to microgrid *k* at time *t* (Yuan/kWh)
$P_{MGi - MGk}^{t}$: The amount of electricity sold from microgrid *i* to microgrid *k* at the time *t* (kWh)
$P_{el}^{j,t}$: The output power of hydrogen fuel cell *j* at time *t* (kW)
$P_{DG}^{i,j,t}$: The output power of the DG *j* in microgrid* i* at time *t* (kW)
$P_{grid}^{t}$: The power provided by the external power grid at time *t* (kW)
$P_{load}^{t}$: The load of the system at time *t* (kW)
$P_{loss}^{t}$: The network loss of this system at time *t* (kW)
$P_{i,genj}^{t}$: The output power of the generator *j* in microgrid *i* at time *t* (kW)
$E^{i,j,t}$: The amount of electricity that the battery *j* can store in microgrid *i* at time *t* (kWh)
$P_{ch\arg e}^{i,j,t}$: The charging capacity of the battery *j* in microgrid *i* at time *t* (kW)
$P_{disch\arg e}^{i,j,t}$: The discharge capacity of battery *j* in microgrid *i* at time *t* (kW)
$SOC^{i,j,t}$: The remaining power of battery* j* in microgrid *i* at time *t*
$DOD^{i,j,t}$: The discharge depth of battery *j* in microgrid *i* at time *t*
